# Soft Skins With Reversible Thickness Morphing: Materials, Mechanisms, and Applications

**DOI:** 10.1002/adma.74369

**Published:** 2026-08-01

**Authors:** Oliver Ozioko, Chiamaka Akah, Ravinder Dahiya

**Affiliations:** ^1^ School of Engineering and Built Environment University of Derby Derby UK; ^2^ Bendable Electronics and Sustainable Technologies (BEST) Group Department of Electrical and Computer Engineering Northeastern University Boston Massachusetts USA

**Keywords:** computer science, contact mechanics, electronic skin, flexible electronics, haptic technology, hybrid system, morphing, programmable matter, soft robotics, tactile sensor

## Abstract

Soft skins with reversible thickness morphing represent a distinct and underexplored class of adaptive material interfaces. Unlike conventional soft actuators that achieve motion through bending, elongation, or twisting, these systems enable out‐of‐plane deformation, producing localized protrusion, retraction, and programmable contact mechanics without rigid support structures. This review reframes thickness modulation not merely as an actuation outcome, but as a material–architecture strategy that couples energy transduction, geometry, and compliance to enable new modes of haptic interaction, morphological adaptation, and operation in confined or unstructured environments. We present a comprehensive synthesis of thickness‐morphing soft skins, covering actuation stimuli, material platforms, structural architectures, fabrication strategies, modeling frameworks, and system‐level integration. Particular emphasis is placed on hierarchical elastomer composites, origami‐ and kirigami‐inspired designs, electrohydraulic and multimodal hybrid systems, and emerging data‐driven control approaches that expand the functional design space. Despite rapid progress, key challenges remain in durability under cyclic loading, energy efficiency and autonomy, scalable manufacturing, and integration of sensing, actuation, and computation. Addressing these challenges will enable self‐powered, fault‐tolerant, and computationally intelligent soft skins capable of embodied perception and safe autonomous operation, positioning thickness morphing as a foundational design axis for next‐generation haptics and soft robotic systems.

## Graphical abstract

Evolution of electronic skin (e‐skin) technologies toward adaptive, multifunctional soft skins. Phase I highlights early rigid and discrete sensory interfaces. Phase II shows the transition toward flexible, stretchable, and large‐area e‐skin. Phase III captures the emergence of computational e‐skin. Phase IV represents advances in active e‐skin, where embedded actuators enable reversible out‐of‐plane deformation, thickness morphing, and dynamic surface reconfiguration, supporting haptic feedback, shape display, and adaptive interaction with unstructured environments.

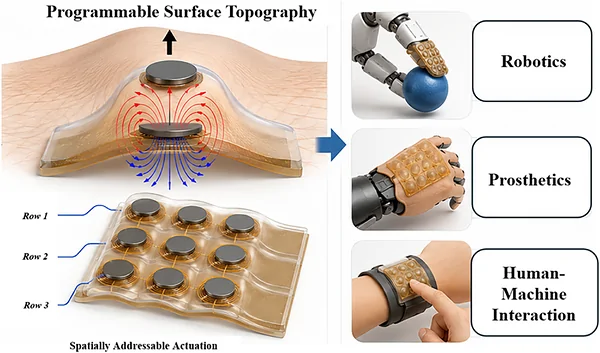

## 1 Introduction

Soft actuators and artificial muscles have emerged as key enabling technologies for systems that require intrinsic compliance, adaptability, and mechanical safety [1, 2]. Their ability to undergo large, reversible deformation allows conformal contact with irregular surfaces, safe interaction with delicate objects, and robust operation in unstructured or dynamically changing environments. As a result, soft actuation has been widely explored in soft robotics, biohybrid systems, wearable devices, and biomedical technologies, where rigid mechanisms are either impractical or unsafe [3, 4, 5]. Over the past two decades, the field has diversified rapidly, with reported deformation modes including bending [6, 7], twisting [8, 9], elongation [10], coiling [11, 12], and out‐of‐plane motion [13, 14].

Among these modes, out‐of‐plane deformation, realized through shrinkage and expansion in thickness, offers a set of capabilities that are qualitatively different from those achievable through purely in‐plane actuation. By enabling volumetric transformation normal to a surface, thickness modulation allows soft systems to dynamically change profile, contact geometry, and mechanical impedance while remaining thin and compliant in their undeformed state. These capabilities are particularly advantageous in confined or unpredictable environments, where changes in cross sectional geometry can determine accessibility, stability, or functionality. For example, minimally invasive surgical tools and microrobots must shrink to traverse narrow biological pathways and subsequently expand to perform localized manipulation [15]. Search‐and‐rescue robots operating in collapsed or cluttered environments must squeeze through debris before expanding to brace, anchor, or exert force [16, 17]. Industrial inspection robots navigating pipelines or ducts must dynamically adapt their diameter to pass through constrictions and resume sensing or cleaning operations. Similarly, deployable aerospace structures rely on compact stowage followed by controlled out‐of‐plane expansion to achieve functional geometries in orbit [18, 19].

From a materials and mechanics perspective, reversible thickness morphing introduces an independent deformation axis that is fundamentally distinct from planar stretchability. Here, thickness morphing refers to reversible out‐of‐plane surface‐normal deformation, producing localized protrusions (positive out‐of‐plane displacement) or retractions (negative out‐of‐plane displacement). Unlike conventional in‐plane actuation, which primarily redistributes surface area and modifies lateral geometry, thickness morphing directly couples material or structural deformation to surface‐normal displacement, contact force, and local stiffness. For clarity, we distinguish two sub‐classes of thickness‐morphing mechanisms. Material‐thickness‐dominated modes (Figure 1a1), which involve true volumetric deformation along the thickness direction (e.g., hydrogel swelling and pneumatic inflation), and geometry‐driven modes (Figure 1a2) which produce out‐of‐plane displacement through folding, bending, or structural design (e.g., kirigami, origami, and domed architectures) without necessarily changing material thickness. Thickness‐direction deformation enables soft skins to function simultaneously as interfaces, structures, and actuators, embedding mechanical intelligence directly within the material architecture and providing a unifying framework for comparing diverse actuation technologies and identifying common scaling limits across materials, architectures, and applications. Thickness‐direction deformation enables soft skins to function simultaneously as interfaces, structures, and actuators, embedding mechanical intelligence directly within the material architecture. Recognizing thickness as a primary design dimension therefore provides a unifying framework for comparing diverse actuation technologies that might otherwise appear unrelated, and for identifying common scaling limits that govern performance across materials, architectures, and applications.

**FIGURE 1:**
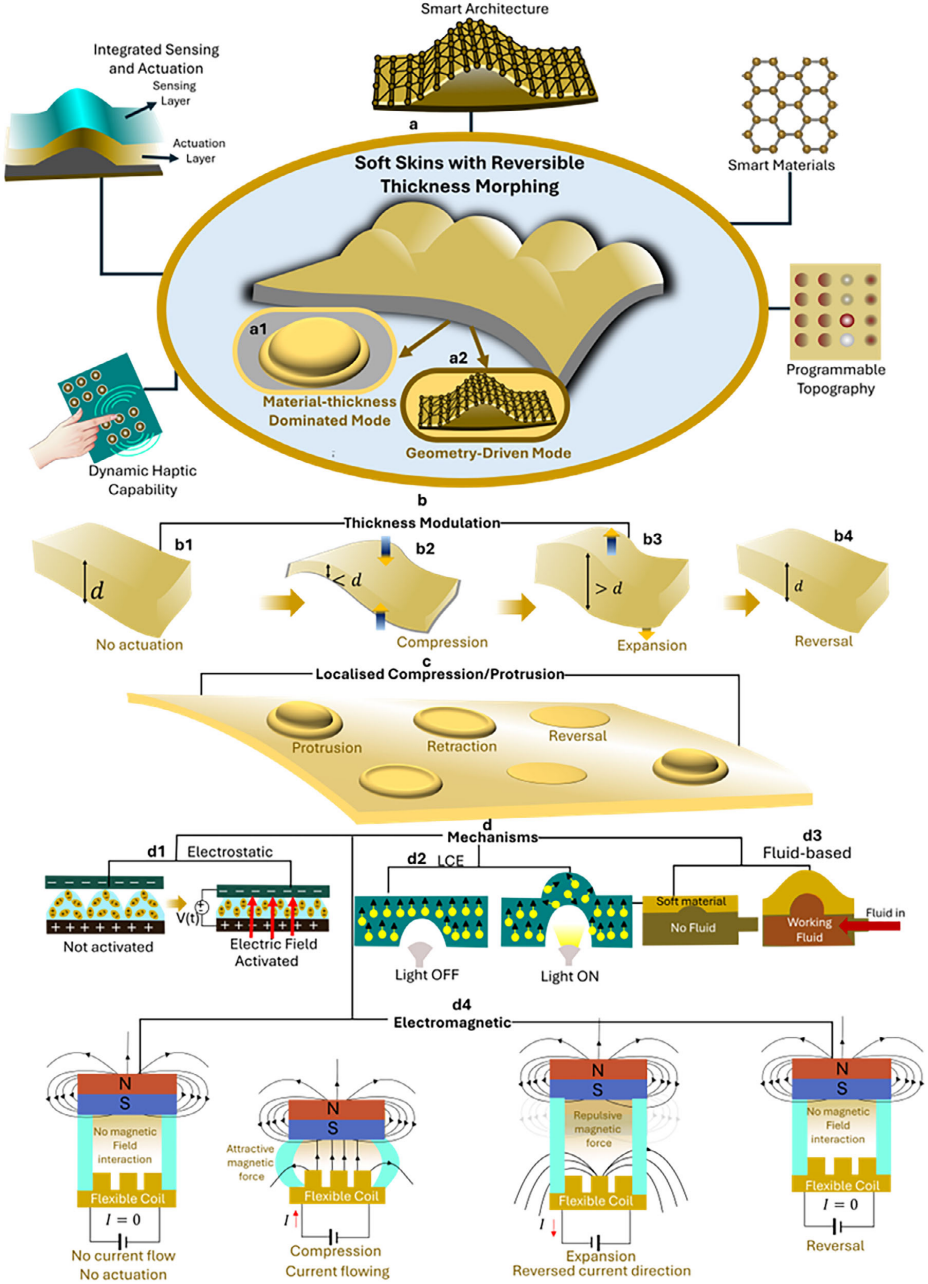
The concept of soft skin that expand and contract in thickness. (a) Key characteristics of soft skin that can expand and contract in thickness. (b) Thickness modulation. (c) Localized compression or protrusion. (d) Example of mechanisms used in soft skins to achieve compression, expansion, and reversal to original shape, including electrostatic, LCE, fluid‐based, and electromagnetic actuation mechanisms.

Building on advances in soft materials, including elastomers, hydrogels, liquid crystalline elastomers (LCEs), soft magnetic composites, and fluidic systems, recent research has enabled a new class of soft skins that reversibly shrink and expand in thickness [20]. These systems differ fundamentally from conventional soft actuators, which typically produce motion along a principal axis through elongation, bending, or twisting. Instead, thickness‐morphing soft skins achieve controlled out‐of‐plane deformation, leading to localized surface protrusion, retraction, and programmable topography, as shown in Figure 1. By translating material‐level actuation into spatially distributed thickness change, these skins enable dynamic haptic interfaces, adaptive surface textures, and morphing envelopes that can respond autonomously to environmental constraints. In haptic systems, precise control of normal displacement allows realistic tactile feedback without bulky mechanisms. In adaptive surfaces and camouflage, reversible thickness modulation enables dynamic texture generation and shape reconfiguration. In confined robotic systems, variable geometry enhances locomotion, anchoring, and interaction with complex surroundings.

To achieve reversible thickness modulation, researchers have explored a broad range of soft material platforms combined with diverse actuation stimuli (Figure 1). Electrostatic [21], electromagnetic [22], fluid‐driven [23], thermal, optical, osmotic, and biohybrid mechanisms have all been demonstrated to produce localized out‐of‐plane deformation when appropriately constrained. In many cases, architectural strategies such as origami‐ and kirigami‐inspired patterning, multilayer composites, and hierarchical morphologies amplify small material strains into large surface‐normal displacements. Hybrid and multimodal systems further combine complementary actuation principles to balance stroke, force, bandwidth, efficiency, and controllability [24]. Collectively, these developments signal a shift from passive, compliant layers toward active, perceptive, and reconfigurable soft skins that integrate sensing, actuation, and control within a unified material system.

Despite rapid progress and compelling laboratory demonstrations, significant challenges remain in translating thickness‐morphing soft skins into robust, autonomous, and field‐deployable technologies. Cyclic volumetric deformation imposes stringent demands on material durability and interfacial integrity. Many high‐performance systems rely on external power sources, high voltages, or fluidic tethers that limit autonomy. Scalable fabrication of large‐area or high‐density skins remains nontrivial, and integrating sensing, computation, and closed‐loop control without compromising softness or reliability continues to pose fundamental design challenges. Addressing these issues requires a materials‐centric perspective that goes beyond individual actuation mechanisms to consider how geometry, energy transduction, transport phenomena, and multifunctional integration jointly govern performance.

While numerous reviews have addressed soft actuation technologies in general, covering dielectric elastomer actuators (DEAs), hydrogel‐based systems, pneumatic devices, and soft conductors, most focus on actuators that deform primarily in‐plane or treat thickness change as a secondary effect [4, 25, 26, 27, 28, 29, 30, 31]. Very few reviews have systematically examined soft skins that explicitly exploit reversible thickness modulation as a primary functional mechanism, nor have they unified these systems under a common conceptual framework that emphasizes geometry, materials architecture, and interface‐level functionality. In contrast, the present review consolidates thickness‐morphing soft skins as a distinct class of adaptive material systems. By organizing the literature around surface‐normal deformation rather than actuation stimulus alone, this review bridges materials science, mechanics, and interface engineering to reveal how thickness change enables localized haptics, dynamic morphing, and adaptive contact mechanics across scales.

Through this synthesis, the review delineates the state of the art in soft skins that enable dynamic thickness modulation, establishes thickness morphing as an independent materials design axis for adaptive interfaces, and charts pathways toward autonomous, perceptive, and robust systems. In doing so, it identifies the key materials, architectural, and integration strategies required to transform laboratory demonstrations into practical, deployable soft skins. The remainder of this article is organized as follows: Section 2 discusses biological and functional inspirations for thickness‐changing mechanisms and gives an overview of the Evolution of e‐skin research. Section 3 classifies actuation stimuli capable of driving reversible out‐of‐plane deformation. Section 4 examines material platforms that encode and regulate thickness morphing. Section 5 addresses integrated and hybrid system architectures. Section 6 evaluates the reviewed actuation technologies using key reported performance metrics. Section 7 highlights some of the representative applications of thickness‐morphing soft skins. Section 8 synthesizes key challenges and outlines future directions toward self‐powered, computationally intelligent, and fault‐tolerant soft skins capable of embodied perception and safe operation in unstructured environments.

## 2 Evolution of E‐Skin Research

This section focuses on the technological evolution of artificial and morphing skin systems, emphasizing advances in materials, actuation, and system integration rather than biological skin anatomy, which is discussed elsewhere [32, 33]. The concept of “artificial skin” in engineering has evolved from rigid electronic surfaces toward compliant, multifunctional materials that mimic the adaptability of biological skin. As may be noted from Figure 2, the advances in e‐skin technology could be broadly categorized into four phases, described below. Although these phases started at different times, they now run in parallel and advancing the e‐skin research toward truly biological skin, not just in terms of morphology, but also in terms of embedded functionality. Additionally, there have been various other fundamental studies on biological skin that continued in parallel to the technological advances, these helped define perceptual limits of biological skin (particularly, human skin) and influenced the designs of e‐skins in different ways. The key findings of these studies, particularly in terms of perceptual limits of thickness expansions and shrinkage, are discussed at the end of this section and summarized in Table 1.

**FIGURE 2:**
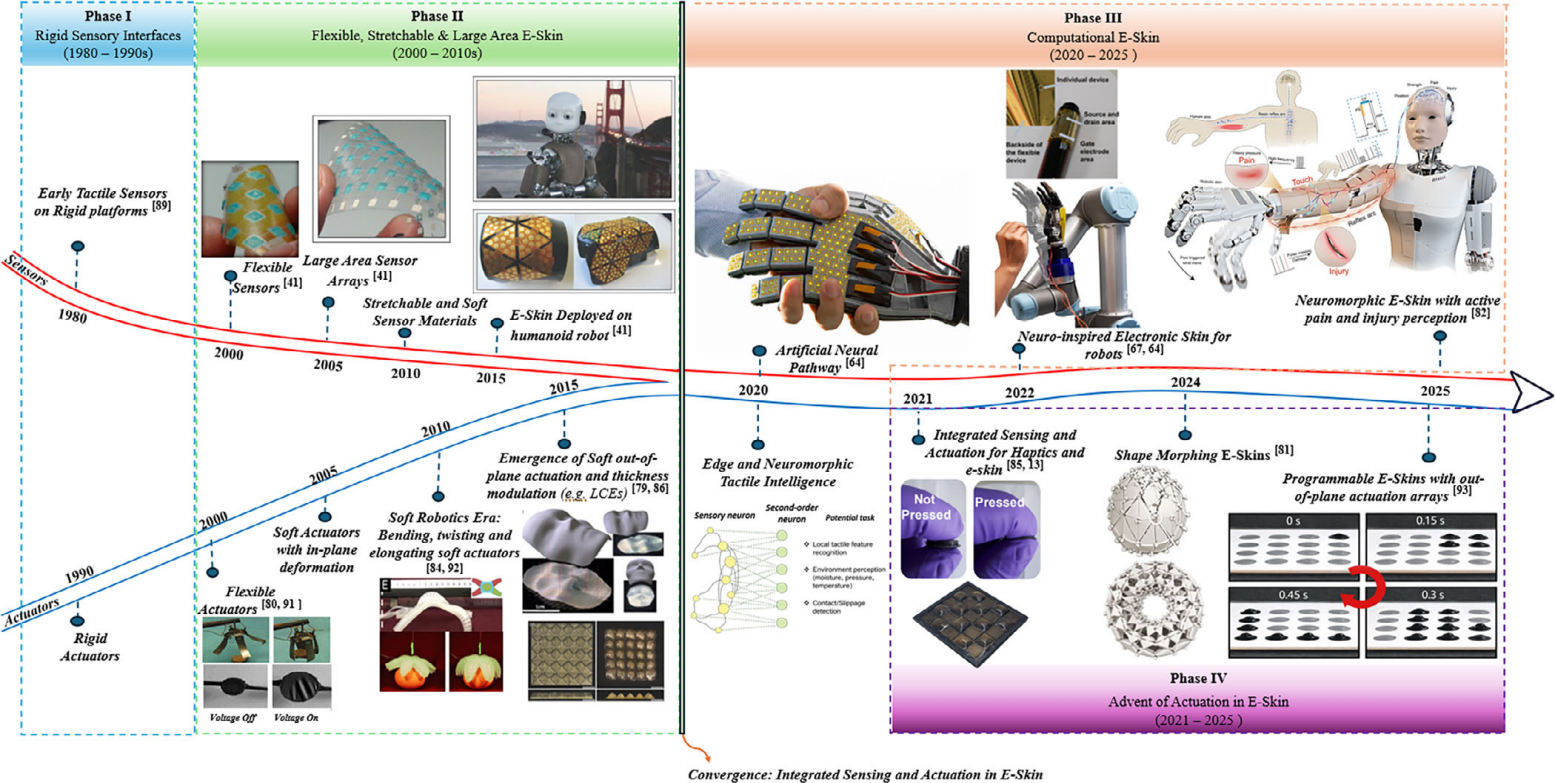
Evolution of e‐skin technologies toward adaptive, multifunctional soft skins. Phase I highlights early rigid and discrete sensory interfaces, primarily focused on basic touch detection. Phase II shows the transition toward flexible, stretchable, and large‐area e‐skin enabled by soft materials, printed electronics, and conformal sensor arrays. Phase III captures the emergence of computational e‐skin, integrating dense sensing, signal processing, and data‐driven perception for intelligent interaction. Phase IV represents recent advances in active e‐skin, where embedded actuators enable reversible out‐of‐plane deformation, thickness morphing, and dynamic surface reconfiguration, supporting haptic feedback, shape display, and adaptive interaction with unstructured environments. Representative examples are adapted and compiled from prior works [13, 41, 64, 67, 79, 80, 81, 82, 83, 84, 85, 86, 87, 88, 89, 90, 91, 92, 93]. Copyright permissions and licensing for reproduced and adapted elements have been obtained from the respective copyright holders.

**TABLE 1:** Design requirements and perceptual constraints for soft skins with reversible thickness morphing.

Parameters	Requirements
Force output [96, 100, 101]	∼ 5 mN is sufficient to excite about 90% of SA‐I and FA‐I mechanoreceptors. ∼ 60 mNcm^−2^ adequately stimulates the finger mechanoreceptors. Certain actuators (e.g., Lorentz force + jamming composites in [100]) have shown up to 4.6 N output while retaining shape morphability.
Spatial resolution [96, 102]	Two‐point discrimination on the finger is about 2 to 4 mm. Threshold on the upper arm is about 45 mm Array pitch should match the finest two‐point discrimination on the target site. Perceivable diameter of a raised feature about 40 µm for a feature height about 8 µm.
Physical displacement in thickness [96, 103, 104]	About 10 to 50 µm detectable indent across areas of the palm. About 100 µm deemed sufficient from finger to upper arm. Actuators such as 3D‐printed zipping electrostatics [103] and planar coil‐based Lorentz actuators [104] have demonstrated vertical strokes of 0.8–2 mm under modest voltage/current stimuli. Static displacement resolution is about 0.02 cm.
Visible displacement in thickness	Depends on lighting, contrast, and viewing distance. Under typical conditions, surface height variations of ∼10–50 µm can be visually discernible, while smaller displacements are optically imperceptible without magnification [105, 106, 107]
Flexibility and stretchability [102]	About 15%, similar to human skin.
Bi‐directional actuation [108, 109]	Actuator should provide bi‐directional motion, shrinking and expanding in response to external excitation. This has been demonstrated using symmetric coil configurations or bistable morphologies (e.g., dome‐shaped DEA [108] or magneto‐buckled forms [109]).
Power requirements	Low power; operating voltage less than 5 V preferred for wearables. Consider self‐powered solutions
Vibration frequency range [96, 110]	Relatively low frequency about 5 to 50 Hz excites FA‐I receptors in dermal papillae of the fingertip (about 70 to 140 cm^−2^). Low frequency below 5 Hz can excite SA‐I receptors in the epidermis of the fingertip (about 70 to 140 cm^−2^). High frequency about 40 to 1000 Hz excites FA‐II receptors in dermal and subcutaneous tissue (about 20 cm^−2^), with peak sensitivity typically above about 64 Hz.
Shape modulation ratio [100, 111]	Actuators must achieve a compressibility or expansion ratio ą 50% of their original form to support collapsibility, deployability, or adaptive surface morphing [100]. Some designs (e.g., SEAM‐II [111]) show ą 60% compression and fast relaxation under external fields.
Mechanical robustness [103, 112]	Cyclic durability > 10 000 cycles are desired for repeated actuation without fatigue. Devices such as HAXELs [103] and ferrofluid‐based voice coils [112] exhibit minimal degradation over thousands of cycles in wet, dry, or dynamic settings.
Environmental resilience [113]	Functionality should remain stable under varied humidity, temperature (10°C to 60°C), and fluidic exposure. Waterproof composites and encapsulated magnetic interfaces (e.g., ecoflex‐encased LM coils) show resilience in different environments [113].
Compactness and conformability [114, 115]	Planar thickness < 1 mm and lateral span < 10 mm are ideal for integration in mobile robots or e‐skin arrays. Many shrinkable actuators are fabricated using printing or molding techniques to yield 0.5–0.8 mm form factors
Sensing compatibility [116, 117]	Preference for integration with embedded sensors to enable feedback‐rich control. Some systems permit self‐sensing under deformation.

### 2.1 Phase I—Rigid Sensory Interfaces

The earliest attempts at skin‐like interfaces emerged between the 1970s and 1990s, when rigid substrates such as silicon and metallic foils were used to host arrays of discrete sensors and electrodes for pressure or strain detection [32, 33, 34]. These rigid skins were primarily developed for robotic manipulators and prosthetic systems, emphasizing sensing accuracy and signal fidelity over mechanical compatibility. Their structural stiffness, however, imposed significant mechanical mismatch with soft or curved surfaces, reducing durability and limiting tactile realism.

### 2.2 Phase II—Flexible, Stretchable, and Large Area E‐Skin

The transition from rigid to flexible electronic skins (e‐skins) began in the early 2000s, enabled by advances in thin‐film electronics, polymer substrates, and stretchable interconnects [35, 36, 37, 38]. Printed circuit boards using polyimide or Polyethylene Terephthalate (PET) backings and serpentine metallic traces introduced mechanical compliance without compromising electrical function [39, 40, 41]. These flexible skins could bend and conform to curved robotic or prosthetic geometries, representing a major leap toward tactile sensing over large and complex surfaces. Early demonstrations integrated pressure, strain, and temperature sensors to emulate limited aspects of human touch [36, 42, 43, 44, 45, 46]. However, such systems primarily exhibited in‐plane flexural compliance rather than true stretchability, as deformation remained largely in‐plane. The mechanics were dominated by geometrical flexibility rather than intrinsic material softness. A transformative phase began with the emergence of stretchable and soft electronic materials around 2010, which established the foundation for today's soft skins [47, 48, 49, 50, 51]. The introduction of elastomeric matrices (e.g., PDMS, ecoflex, and SEBS) combined with compliant conductors such as liquid metals, conductive hydrogels, carbon nanotube (CNT) percolation networks, and silver nanowires enabled high stretchability and conformability [52, 53, 54]. Flexible tactile sensor arrays with ultrathin polymeric substrates and integrated sensing circuits, bridging materials and microelectronics were demonstrated [55, 56, 57].

### 2.3 Phase III—Computational E‐Skin

The handling of large data generated by the sensors, particularly for large areas implementations such as whole‐body tactile sensing for humanoids, has recently attracted researchers’ attention toward computational approaches such as edge computing, near‐sensors computing, and the use of artificial intelligence (AI) algorithms [36, 58, 59, 60, 61, 62, 63] as well as the ways to implements them in hardware using devices and circuits that can provide some neuron‐like functions [64, 65, 66]. Focusing on the computational aspect of the skin and the associated peripheral nervous systems (PNS) that efficiently process the tactile data, this new dimension of e‐skin research aims to mimic the biological tactile neural pathways for preliminary perception capability to decrease the cognitive load on their central control units. This is analogous to the PNS complementing the functionality of the central nervous system (CNS) in humans. A detailed discussion on the computational aspect of e‐skin, various building blocks of the tactile neural pathways and the integration that could imitate their functionality are discussed in previous review articles [67, 68].

### 2.4 Phase IV—Advent of Actuation in E‐Skin

The above advances in e‐skin paralleled the rapid rise of soft robotics, which demanded actuators and sensors capable of co‐deformation with soft bodies. The growing demand for soft actuators that go beyond movement to provide haptic feedback has accelerated the convergence of actuation and sensing functions. As a result, e‐skins evolved from passive, sensing‐only layers to multifunctional soft interfaces capable of active deformation, distributed sensing, and dynamic interaction with the environment [59, 69, 70, 71]. Throughout this evolution, progress in smart materials has been the defining driver. As an example, electroactive polymers (EAPs) introduced electric‐field‐driven deformation with high strain and silent actuation, paving the way for DEAs and ionic polymer–metal composites (IPMCs) [72]. Likewise, stimuli‐responsive hydrogels expanded the range of actuation stimuli by exploiting reversible swelling under temperature, pH, or ionic gradients [27, 73] LCEs offered programmable anisotropy, linking molecular orientation with macroscopic shape change and providing a route to reversible out‐of‐plane deformation [74]. In parallel, magneto‐responsive and photo‐responsive materials enabled remote or spatially selective activation [25, 75]. Collectively, these material innovations shifted artificial skins from static sensors toward adaptive, shape‐changing, and thickness‐modulating interfaces.

In the last decade, research on soft skins has matured into a multidisciplinary field uniting materials science, mechanics, electronics, and intelligence. Modern systems combine dense sensor–actuator networks, self‐healing elastomers, and embedded computational control, moving toward truly autonomous behavior [13, 76, 77]. In particular, thickness‐modulating skins, capable of reversible expansion and shrinkage, normal to their surface, represent the evolutionary trajectory [13, 78]. They embody the convergence of soft actuation, sensing, and morphing achieved through the co‐development of smart materials, additive manufacturing, and integrated feedback control. These advances point toward the next frontier: autonomous soft skins capable of self‐powered operation, embodied perception, and adaptive morphogenesis in unstructured environments [58, 59, 64, 69].

### 2.5 Perceptual Limits of Movements in the Skin

The biological skin is a dense network of somatosensory receptors (e.g., mechanoreceptors, thermoreceptors, and nociceptors), many of which are tightly coupled with muscles to collectively enable perception of the external environment [94, 95]. These receptors have served as the biological basis for the development of various morphological and functional features of tactile interface reported in past. Consequently, developing intelligent human–machine interfaces (iHMIs) requires a fundamental understanding of the mechanisms underlying human touch [96, 97]. This is also important in context with this review as the dynamic range and adaptation characteristics of receptors in the biological skin could influence the design of actuators intended to produce perceptible haptic feedback. For example, in unstructured and constrained environments, where actuators must interact with irregular surfaces, variable loads, and dynamic contacts, the spatial distribution, frequency tuning, and adaptation rates of human mechanoreceptors enable the skin to discriminate between transient and sustained stimuli while preserving perceptual stability. Translating these attributes into artificial soft skins involves designing materials and architectures capable of modulating stiffness, curvature, and contact force in response to both static and dynamic inputs.

Mechanoreceptors in biological skin respond to mechanical pressure and vibration, thermoreceptors to temperature changes, and nociceptors to painful stimuli, transmitting the corresponding sensory information to the brain through afferent neural pathways [32, 67, 98]. The mechanoreceptors are classified into two types: slowly adapting receptors (SA‐I and SA‐II), which respond to static or quasi‐static stimuli by producing sustained discharge, and fast adapting receptors (FA‐I and FA‐II), which respond to transient, dynamic stimuli such as vibrations [96, 99]. FA‐I receptors are sensitive to low‐frequency stimuli (5–50 Hz), enabling texture discrimination and fine manipulation, while FA‐II receptors detect higher‐frequency vibrations (up to 400 Hz) [99]. FA‐type response principles inform the development of actuators capable of rapid, transient contact‐driven adjustments, while SA‐type behavior inspires systems that maintain stable deformation and sustained contact sensing—essential for continuous grip or posture control under complex loading conditions. These biologically inspired strategies are being embedded at the material, structural, and control levels of soft actuator design, enabling systems that not only deform out‐of‐plane but also adapt their morphology intelligently to environmental and task‐specific constraints.

Unlike rigid actuators, soft systems must maintain functional integrity under uncertain terrain, fluctuating loads, and dynamically changing geometries. High reversible displacement, shape adaptability, and mechanical resilience are critical. Actuators designed for navigation through narrow passages or deformable environments must achieve large, recoverable strain while resisting fatigue from repeated impact, torsion, or environmental exposure. The materials must also exhibit low power demand, environmental sealing, and fatigue resistance, ensuring sustained autonomy in field‐deployable or wearable applications. Recent architectures based on Lorentz‐force actuation and electrostatic zipping have demonstrated substantial out‐of‐plane displacement and force output with minimal electrical input, making them promising candidates for energy‐limited systems [100]. Equally important is the integration of embedded sensing for proprioceptive feedback. By co‐fabricating strain gauges, capacitive sensors, or chromotropic (color‐shifting) layers within the actuator, soft skins can sense their own deformation and adjust accordingly, providing closed‐loop adaptive behavior. Such systems are precursors to expressive actuation, where materials physically respond to both external stimuli and user intention.

Beyond mechanical durability, soft skins must respect the physiological and perceptual thresholds of human tactile sensation [94]. The minimum detectable indentation on human skin lies between 10 and 50 µm, depending on receptor density and body location. Indentations below 10 µm are generally imperceptible, while those exceeding 50 µm may cause discomfort or pain. Likewise, spatial resolution, the minimal separation distance at which two stimuli can be distinguished, is critical when designing actuator arrays. The two‐point discrimination (2PD) threshold, which defines spatial resolution, varies across body regions: approximately 1–2 mm at the fingertips, 8–15 mm on the forearm, and up to 40–50 mm on the upper arm [94]. Actuator design must therefore align array spacing and deformation amplitude with these regional thresholds to produce perceptible yet comfortable haptic cues. These perceptual limits, summarized in Table 1, provide a quantitative framework for designing bio‐congruent soft skins that can effectively interface with human users while maintaining safe, expressive, and energy‐efficient operation in real‐world environments.

The out‐of‐plane indentations of braille have long enabled communication in blind people. For richer haptic interaction in such rehabilitation applications and others such as tactile displays, researchers have explored both single‐element soft actuators and large‐area actuator arrays capable of generating localized tactile stimuli. From a biological perspective, an ideal tactile display should have micrometer‐scale spatial resolution, kilohertz‐level bandwidth, and millimeter‐scale out‐of‐plane displacement for each tactile element. In practical terms, this would correspond to actuator arrays on the order of 10^4^ × 10^4^ elements within a 1 cm^2^ area, operating at frequencies approaching 1 kHz with displacement amplitudes of approximately 1 mm per element [118]. At present, no existing actuation technology approaches this combination of resolution, bandwidth, and displacement, highlighting the need for fundamentally new materials, architectures, and engineering strategies.

Selecting appropriate soft actuator technologies to enable dynamic out‐of‐plane deformation in soft skins requires a multidimensional evaluation framework, particularly for applications involving interactive haptic feedback and operation in unstructured or constrained environments. Unlike rigid actuators that are typically assessed in terms of mechanical output, efficiency, and precision, soft actuators must be evaluated across a broader set of criteria, including mechanical compliance, adaptability to complex geometries, safety during human interaction, fabrication scalability, and long‐term sustainability. These considerations become especially critical for shrinkable and expandable out‐of‐plane configurations, where actuators must maintain functional performance under large geometric deformation, repeated cycling, and environmental uncertainty. The next section presents some of the technologies that enable such controlled deformations.

## 3 Actuation Stimuli for Out‐of‐Plane Thickness Morphing

Reversible out‐of‐plane thickness morphing in soft skins can be achieved through a diverse range of actuation stimuli that convert externally supplied energy into localized mechanical deformation. Increasing attention has therefore been directed toward identifying and optimizing physical transduction mechanisms capable of generating controlled shrinkage and expansion normal to a surface while remaining compatible with soft, compliant substrates. Widely explored approaches include electrostatic, electromagnetic, fluid‐driven, thermal, and light‐responsive actuation, each offering distinct advantages and limitations in terms of achievable stroke, force output, response speed, energy efficiency, and controllability [119].

The suitability of a given actuation stimulus is inherently application dependent. For example, electrostatic systems such as DEAs can deliver fast response and large strain but typically require kilovolt‐level driving voltages, limiting their practicality in wearable or battery‐powered platforms [120]. Fluid‐driven actuators can generate large forces and millimeter‐to‐centimeter‐scale out‐of‐plane strokes but often rely on external pressure sources or pumps that increase system complexity. Magnetic‐ and light‐driven actuators offer wireless and remote‐control capabilities, which are particularly advantageous in sealed, confined, or access‐limited environments, albeit with trade‐offs in spatial precision or integration complexity.

This section surveys the principal actuation stimuli and transduction strategies used to realize soft actuators capable of reversible out‐of‐plane displacement for haptic interaction and deployment in unstructured or constrained environments. While these mechanisms are frequently implemented using specific material systems, the intrinsic material properties that encode deformation, such as anisotropy, phase transitions, solvent transport, and so forth, are treated separately in Section 4 to avoid redundancy and enable systematic comparison.

### 3.1 Electrostatic Actuation

Electrostatic actuators generate motion through the electric forces arising when an electric field is applied between two compliant electrodes. Their relatively simple structure, low mass, and compatibility with soft and flexible materials make them particularly attractive for miniaturized, high‐resolution iHMIs and soft skins capable of out‐of‐plane deformation [118]. When appropriately architected, electrostatic actuators can produce—localized vertical displacement, vibration, or thickness modulation, enabling perceptible haptic feedback upon contact with the human skin.

Among electrostatic technologies, DEAs are one of the most widely explored technologies for tactile feedback generation [108, 121]. A typical DEA consists of a thin elastomer membrane coated on both sides with compliant electrodes. When a high voltage is applied, electrostatic (Maxwell) stress compresses the membrane thickness while inducing in‐plane expansion, resulting in controllable out‐of‐plane deformation [108]. Owing to their fast response and large strain capability, DEAs have been extensively investigated for haptic interfaces and tactile displays. Several strategies have been proposed to amplify the out‐of‐plane displacement of DEAs for haptic interaction. These include the haptic display based on compliant liquid DEAs, combining electrostatic attraction with hydraulic amplification [122]. In this work, a liquid dielectric, encapsulated within a compliant pouch, experiences dynamic pressure variation when voltage is applied across opposed hydrogel electrodes, enabling tactile feedback with displacements exceeding 2 mm and output forces above 0.8 N. Similarly, a hydrostatically coupled DEA was introduced for fingertip haptics in virtual reality [123]. In this design, an incompressible fluid couples an electromechanically active DEA to a passive membrane in contact with the finger. The hydrostatic coupling transforms in‐plane DEA deformation into a stable, spherical‐cap‐shaped out‐of‐plane protrusion suitable for tactile stimulation.

Microstructural biasing has also been employed to tailor DEA response. For example, a tactile actuator based on a pyramidal microstructured DEA, demonstrated vibration generation in the 100–200 Hz frequency range, with tunable characteristics governed by the microgeometry [124]. Another example is the DEA incorporating a three‐dimensional (3D) silicone dome fabricated via casting, where the polymeric dome acts as a mechanical biasing element to magnify out‐of‐plane stroke [108]. Under voltages up to 3 kV, the actuator achieved a stroke of approximately 2.14 mm, with a resonance frequency near 106 Hz over an operating range of 1–200 Hz.

Material selection plays a critical role in determining the performance of shrinkable and expandable electrostatic actuators. Soft elastomers such as polydimethylsiloxane (PDMS) and ecoflex 00–30 are commonly used due to their low elastic modulus and high dielectric breakdown strength. In this regard, the soft and stretchable electrostatic actuator (∼15% stretchability) capable of generating ∼500 µm displacement and pressures approaching 1 MPa is a suitable example [102]. Systematic studies comparing PDMS and ecoflex membranes of varying thicknesses (50 and 100 µm) revealed that thinner membranes (∼50 µm) yield significantly larger out‐of‐plane displacement, underscoring the importance of thickness optimization for vertical morphing performance. Wearable electrostatic haptic systems have also been demonstrated. For example, a multi‐layered hydrostatically coupled DEA architecture has been shown to enable localized, tunable force feedback across multiple fingers, delivering forces up to 0.7 N at 4 kV [125]. In parallel, twisted and coiled polymer (TCP) actuators capable of producing skin‐stretch sensations have been explored as complementary electrostatic‐driven haptic modalities [126].

Beyond DEAs, electrostatic zipping actuators constitute a distinct and increasingly important class of electrostatic systems that are suitable for shrinkable and expandable out‐of‐plane actuation. Unlike DEAs, which rely on uniform membrane deformation, zipping actuators exploit progressive electrode adhesion under Maxwell stress to convert localized electrostatic attraction into large vertical strokes. This mechanism enables compact form factors and efficient thickness modulation, making zipping actuators well suited for soft skins and tactile displays. A prominent example is the hydrostatically amplified taxel (HAXEL) shown in Figure 3e, which combines stretchable elastomers with high‐permittivity flexible films to achieve both high force and large strain [103]. This shows an array of flexible HAXELs generating blocked forces ranging from 100 to 800 mN, with DC displacements between 100 and 850 µm. The authors further demonstrated scalable designs with diameters from 2 to 15 mm, allowing customization for different haptic and morphing applications. Subsequent work reported a fully printed, 200 µm‐thick, stretchable (up to 50%) HAXEL capable of delivering both static indentation and vibrotactile stimuli [127]. These devices consist of oil‐filled stretchable pouches whose shape is controlled by electrostatic zipping [128], enabling reversible out‐of‐plane deformation with minimal mechanical complexity. A review of cooperative actuators and sensor systems based on DEAs can be found here [24].

**FIGURE 3:**
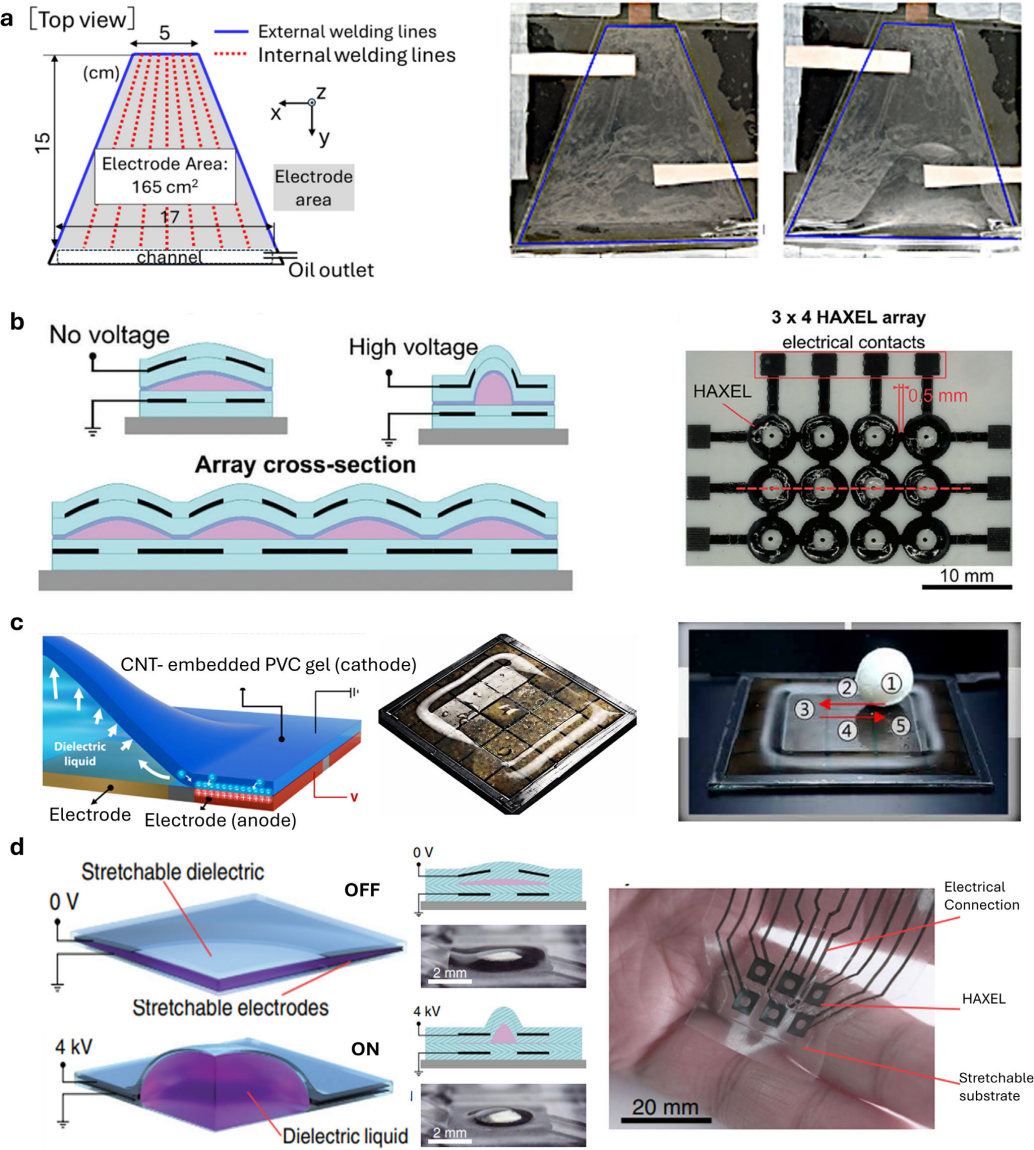
Electrostatic‐based reversible thickness morphing actuators. (a) Optical images illustrating voltage‐induced zipping and fluid redistribution in a single electrohydraulic actuator, resulting in localized out‐of‐plane inflation relative to the unactuated state. Adapted under the terms of the Creative Commons Attribution 4.0 license [129]. Copyright 2025, The Author(s). (b) Actuation principle and array‐level implementation of hydraulically amplified electrostatic actuators (HAXELs), showing electrode zipping under high voltage. Adapted under the terms of the Creative Commons Attribution‐NonCommercial license [130]. Copyright 2025, The Author(s). (c) Large‐area electrohydraulic actuator array demonstrating distributed thickness morphing across a planar surface. Adapted under the terms of the Creative Commons Attribution‐NonCommercial 4.0 International license [85]. Copyright 2024, The Authors; exclusive licensee American Association for the Advancement of Science. (d) Time‐resolved object manipulation and conveyance enabled by programmed spatiotemporal activation of actuator arrays, illustrating dynamic surface reconfiguration. Adapted under the terms of the Creative Commons Attribution‐NonCommercial license [127]. Copyright 2023, The Authors.

Recent advances have further expanded the design space of electrostatic zipping actuators for thickness morphing. The notable examples (shown in Figure 3) include a fan‐shaped electrostatic soft hydraulic actuator (Figure 3a) developed to achieve controlled out‐of‐plane shrinkage [129]. Radial welding lines divided the active chamber into triangular units, promoting sequential zipping from the apex and driving vertical collapse of the top and bottom films. This configuration produced a maximum pressure change of 4.53 kPa at 6 kV—nearly twice that of a rectangular pouch actuator and maintained consistent contraction across orientations from 0° to 90°, demonstrating robustness under varying spatial configurations. Additive manufacturing has also been leveraged to simplify fabrication of these devices and enable dense actuator arrays. In this regard, the fully additive process that integrate liquid dielectric during printing to realize stretchable electrostatic zipping actuators in the form of HAXELs (Figure 3b) is worth noting as it eliminates post‐filling steps [130]. Arrays of 5 mm‐diameter devices with 0.5 mm spacing achieved central displacements of approximately 350 µm at 3 kV and 100 Hz, with performance tunable through filling volume and electrode geometry. Although, in this case, the mechanical output is lower than that of pressure‐filled counterparts, the approach highlights the advantages of embedded‐fluid fabrication for scalable soft skins. The electrostatic zipping geometries can also enable reconfigurable vertical motion. Here, examples include an arch‐shaped electrostatic zipping actuator that converts electrode closure into vertical lift to enable both lateral swing and shrinkable out‐of‐plane displacement suitable for leg lifting in multi‐legged locomotion [131]. Another example exploits perimeter‐initiated zipping in a hydraulically coupled PVC‐gel actuator to generate doming deformation (Figure 3c,d) and can achieve fast morphing to 2.2 mm within 45 ms at 1.5 kV [85]. By repositioning the active electrodes, the dome could be split, enabling reconfigurable 3D surfaces and tactile patterns.

Collectively, these electrostatic and zipping‐based actuators illustrate the versatility of electrostatic mechanisms for achieving reversible thickness morphing in soft skins. While high operating voltages remain a challenge, their fast response, scalability, and compatibility with compliant materials make electrostatic actuators a cornerstone technology for future haptic, morphing, and adaptive soft interfaces.

### 3.2 Electromagnetic Actuation

Electromagnetic actuators operate based on fundamental principles of electromagnetism, including Faraday's law of electromagnetic induction, the Lorentz force law, and the Biot–Savart law, which together describe how electric currents generate magnetic fields and forces [132]. Conventional electromagnetic actuators such as DC motors, AC motors, stepper motors, and voice‐coil actuators, typically comprise rigid electrical and magnetic circuits and are optimized for high force density and precise control [132]. However, their inherent rigidity, bulkiness, and mechanical mismatch with soft substrates significantly limit their suitability for wearable systems, haptic interfaces, and skin‐like robotic surfaces, where flexibility, lightweight construction, and conformability are essential.

To address these limitations, researchers have increasingly explored soft electromagnetic actuators that combine compliant materials with current‐driven magnetic elements to enable deformable and skin‐compatible actuation. By embedding coils, magnets, or magnetically responsive structures within elastomers, textiles, or soft composites, electromagnetic forces can be harnessed to produce reversible out‐of‐plane deformation, localized indentation, and thickness modulation. This section focuses on such soft electromagnetic actuators, both single elements and arrays, designed to provide haptic feedback and adaptive functionality in constrained or unstructured environments.

Early work explored planar and flexible electromagnetic coils fabricated using micro‐ and nanofabrication techniques to enable thin, conformable actuators [13]. The examples include flexible electromagnetic actuator consisting of a magnetic membrane suspended above a flexible induction coil [114]. The actuator operated in both vibrational and non‐vibrational modes, achieving maximum displacements of approximately 651 and 187 µm, respectively. By tuning the driving conditions, the device could deliver perceivable tactile stimuli while maintaining mechanical compliance. Array‐based electromagnetic systems have also been reported to enhance force output and functionality [133]. Here, an array of soft actuators capable of operating in both latching and non‐latching modes is worth noting. Each unit combined an electromagnetic coil, a permanent magnet, a thin ferromagnetic sheet, and a water‐based hydraulic mechanism. In non‐latching mode, the actuator delivered forces up to 1.3 N, while in latching mode, force output increased to 5.2 N when preloaded with 3.7 N of compression. The hybrid electromagnetic–hydraulic architecture used in this work enabled sustained force generation with reduced energy consumption.

Liquid metals have further expanded the design space of soft electromagnetic actuation [134, 135]. A soft robotic structure incorporating liquid metal conductors achieved reversible 3D shape morphing driven by Lorentz forces when electric current flowed through the liquid metal channels [100]. A granular core embedded within a silicone rubber shell provided structural stability through a jamming mechanism when the current was turned off. The actuator demonstrated reversible shape changes with amplitudes exceeding 10 mm, illustrating the potential of liquid‐metal‐based electromagnetic actuation for large out‐of‐plane deformation.

Textile‐based approaches offer another route toward wearable electromagnetic skins. The examples include a fabric‐based electromagnetic actuator fabricated via copper‐mesh hot‐pressing technology on textiles such as cotton, nylon, terylene, and silk [136]. Using this scalable process, the authors realized 3 × 1 and 2 × 2 actuator arrays, highlighting the feasibility of integrating electromagnetic actuation directly into garments or soft surfaces. Similarly, an array of magnetic actuators combining microscale flexible planar coils with magneto‐responsive polymer effectors has been reported [115]. By programming the magnetic properties of the effectors and exploiting nonhomogeneous near‐field magnetic distributions, the actuators achieved lifting, tilting, pulling, and grasping motions, demonstrating multifunctional out‐of‐plane control.

More recent work has focused explicitly on shrinkable and expandable out‐of‐plane electromagnetic actuation for soft skins such as the fully integrated silicone‐based actuator embedded a lightweight copper coil and a small NdFeB magnet within an elastomeric body [137]. The device exhibited clear current‐dependent vertical displacement with attraction mode closing an initial 5 mm gap at 1.5 A, and repulsion mode producing 4.5–4.6 mm of out‐of‐plane expansion at the same current (shown in Figure 4a). Blocking forces reached approximately 0.10 N at 1.5 A and increased to 0.15 N at 2.0 A, with corresponding power consumptions of 2.8 W and 4.7 W. The actuator could tolerate up to 94% tensile strain and operated continuously at 1 Hz. System‐level demonstrations of this work include a walking robot achieving up to 41.7% contraction and 2.1 mm expansion, reaching speeds of approximately 27.6 body lengths per second at 1.5 A and 1.5 Hz.

**FIGURE 4:**
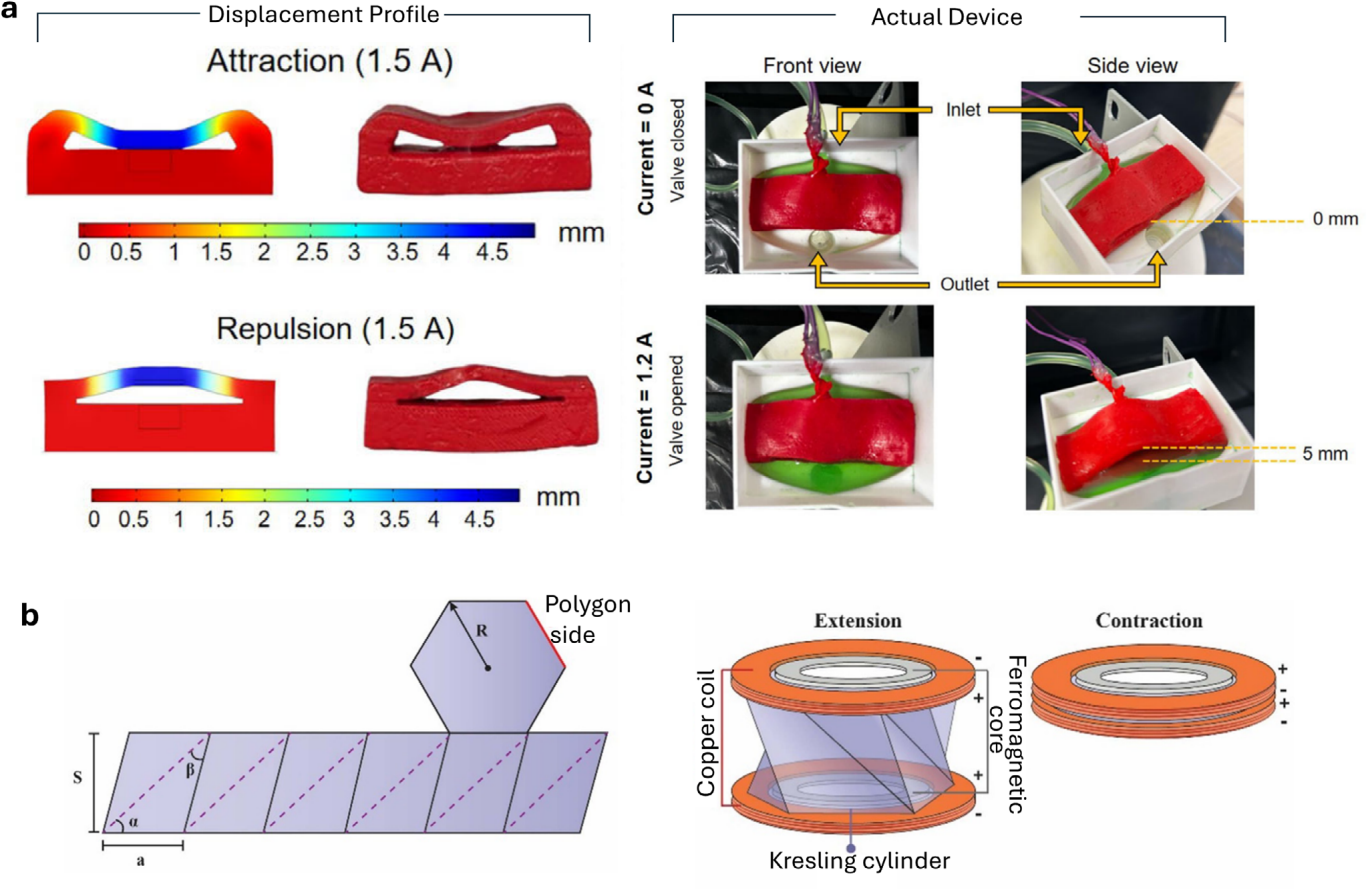
Electromagnetic‐based reversible thickness morphing actuators. (a) Soft electromagnetic actuator operating in attraction and repulsion modes, showing current‐controlled vertical displacement, simulated and experimental deformation profiles, and reversible out‐of‐plane thickness modulation under applied electrical current. Adapted under the terms of the Creative Commons Attribution license [137]. Copyright 2025, The Author(s). (b) Origami‐inspired electromagnetic actuator concepts, including a Kresling‐based structure demonstrating reversible axial contraction and extension driven by embedded electromagnetic actuation, highlighting bidirectional thickness and length morphing through structural geometry. Adapted under the terms of the Creative Commons Attribution 4.0 license [140]. Copyright 2025, The Author(s).

Muscle‐inspired designs have further improved performance efficiency. For example, an elasto‐electromagnetic actuator balanced elastomeric restoring forces against coil–magnet attraction to achieve large stroke at low voltage [138]. The actuator delivered output forces of approximately 210 N per kilogram of actuator mass, with designable contraction ratios up to 60% and operating voltages below 4 V. Characteristic operation around 60 Hz has been demonstrated, along with a bistable motor strategy that reduced energy consumption to 1.2%–38.4% of that required for full‐stroke actuation while preserving displacement. Compact prototypes achieved strokes near 30% and displacements of about 5 mm, enabling autonomous insect‐scale robotic locomotion. Electromagnetic actuation has also been adapted for fingertip haptics [139]. A soft tactile electromagnetic actuator employing a thin compliant diaphragm and compact electromagnetic driver generated perceptible protrusions and forces. The actuator produced a maximum force of approximately 0.40 N at 0.1 Hz with 750 mA input, a peak out‐of‐plane displacement of about 0.63 mm at resonance, and a peak acceleration near 1250 m s^−^^2^. The resonance frequency was around 240 Hz and varied with drive conditions. Step‐response measurements showed a rise time of 44.6 ms to reach 90% of maximum force, while thermal imaging indicated a temperature of approximately 40°C after 100 s at maximum drive, which is well within acceptable limits for short‐duration haptic stimulation [139].

Geometric amplification strategies can be adopted to further enhance electromagnetic thickness morphing. As an example, an origami‐inspired Kresling cylinder with embedded electromagnets converted electromagnetic loading into axial shrink–expand motion [140]. The prototype (Figure 4b) achieved a stroke length of approximately 26 mm for a cylinder radius of 30 mm, using coils with about 220 turns of 0.3 mm wire driven at roughly 3 A and weighs about 36 g. Mechanical characterization indicated an average peak resistance force of about 0.33 N, and parametric studies elucidated coil design trends for improved force generation at fixed mass and dimensions [140].

Finally, theoretical and computational frameworks have supported the systematic design of electromagnetic out‐of‐plane morphing networks. The mechanics‐based modeling has been shown to exhibit a linear relationship between peak normalized displacement and applied electromagnetic forcing in serpentine soft networks [141]. Analytical predictions agreed with finite‐element simulations and experiments within approximately 7% error for maximum displacement. Representative designs employed magnetic fields around 170 mT, network layer thicknesses of ∼5 µm, ribbon widths of ∼90 µm, and substrate thicknesses near 800 µm, enabling voltage‐controlled programming of complex out‐of‐plane surface profiles [141].

### 3.3 Fluid‐Driven Actuation

Fluid‐driven actuators generate force and displacement by converting energy stored in a working fluid, such as air, water, or oil, into mechanical deformation of compliant structures, typically fabricated from elastomers or other flexible polymers [142]. They shrink and expand out‐of‐the‐plane through controlled pressure differentials or volume changes within enclosed chambers, transferring fluid energy into structural deformation (Figure 5). The choice of working fluid depends strongly on application requirements [143]. Compressible fluids, such as air, are commonly used in systems where low stiffness and gentle interaction are desired, whereas incompressible fluids, including water and oil, are preferred for applications requiring higher force density, faster response, or improved positional stability. Although compressible systems often exhibit slower dynamic response, advances in sensing and control have enabled compensation strategies that significantly improve bandwidth and accuracy. This section focuses on fluid‐driven soft actuators capable of reversible out‐of‐plane shrinkage and expansion, particularly those developed for haptic interaction and operation in constrained environments. In these systems, pressure variations within compliant architectures are transformed into localized protrusions, thickness modulation, or muscle‐like axial contraction, enabling large deformation with high force‐to‐weight ratios.

**FIGURE 5:**
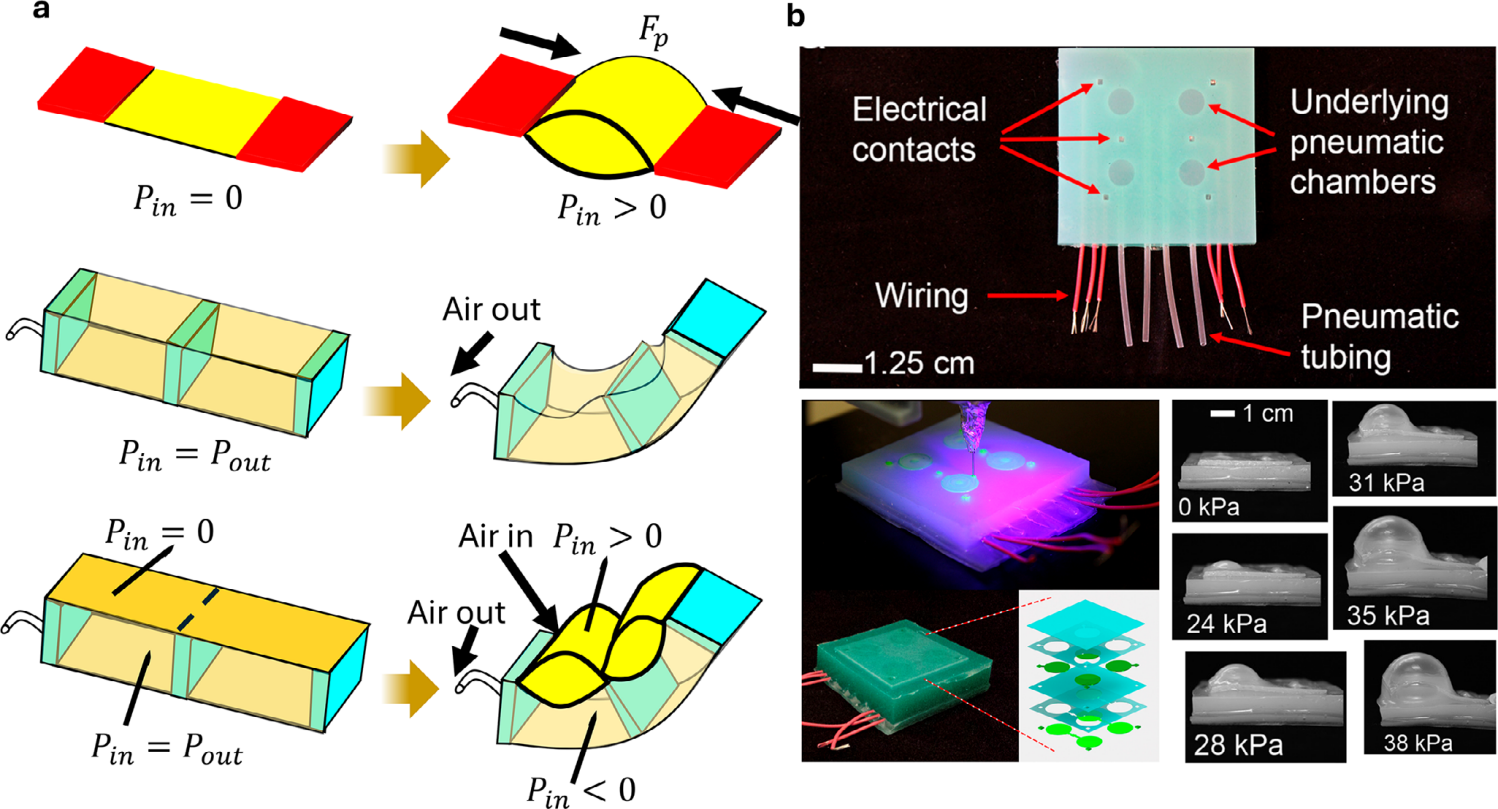
Fluid‐driven actuators. (a) Mechanisms of fluid‐driven actuators. Adapted under the terms of the Creative Commons Attribution 4.0 license [148]. Copyright 2021, The Author(s). (b) A pneumatic haptic display with wells where electrical contacts will be formed, underlying pneumatic chambers, tubing, and electrical wiring. Adapted with permission [149] Copyright 2015, Elsevier Ltd.

A wide range of positive‐pressure fluidic actuators have been reported that achieve out‐of‐plane deformation through chamber expansion. Fiber‐reinforced elastomeric enclosures represent a prominent class, where anisotropic reinforcement converts internal pressure into controlled axial motion. The example include a parallel arrangement of such enclosures which when pressurized up to 103.4 kPa generated a full two‐dimensional (2D) force set, with root‐mean‐square force errors below 1.5 N and maximum errors under 3 N [144]. The derived fluid Jacobian mapping demonstrated programmable axial shrink–extend behavior when modules were configured for longitudinal output. Similarly, a three‐chamber hydraulic actuator, reported for MRI‐guided needle manipulation, exploits water‐filled chambers to generate controlled tilting and locking motions [145]. The system achieved needle navigation accuracy of 0.89 ± 0.31 mm, actuator bandwidth of approximately 1.1 Hz, hydraulic transmission latency of about 160 ms at 1.1 Hz, and static stiffness near 2.337 N mm^−^^1^, illustrating precise chamber‐based out‐of‐plane control.

Bellows‐based architectures provide another route to axial thickness modulation. Here the examples include a pneumatic bellows muscle constructed from rigid plates and compliant hinges contracted under positive pressure [146]. Geometric analysis predicted contraction ratios of approximately 23.9% and 29.3% for two designs, with experimental prototypes achieving contraction around 8.6%, corresponding to direct out‐of‐plane thickness reduction. Compact hydraulic actuators have also been developed for tool manipulation and feeding [147]. For example, a small‐scale soft hydraulic linear actuator coupled a central bellows to passive grippers, using internal pressure as an intrinsic force sensor. The device delivered over 1 N to the tool when fully inflated and produced gripping forces of 2.76 ± 0.22 N, 1.40 ± 0.08 N, and 0.97 ± 0.05 N for tools of 1.25, 1.00, and 0.50 mm diameter, respectively. Force estimation errors were as low as 0.12 N at 0.4 mL and 0.04 N at 0.8 mL, with stroke characterization performed over discrete volume increments that emulate millimeter‐scale feed motion.

Beyond positive‐pressure operation, negative‐pressure (vacuum‐driven) actuators have significantly expanded the design space for out‐of‐plane shrinkage. Vacuum‐sealed origami pneumatic artificial muscles (OV‐PAMs) report up to ∼90% linear contraction and output forces approaching ∼400 N, with some designs achieving large strokes under vacuum levels as low as ∼10 kPa [150]. These results highlight how geometric confinement and folding can sustain high axial force along the out‐of‐plane direction. Similarly, vacuum‐actuated muscle‐inspired pneumatic structures (VAMPs) exploit cooperative buckling of elastomeric beams to achieve ∼40% longitudinal contraction while offering intrinsic fail‐safe behavior, making them particularly attractive for wearable and human‐interactive systems [151].Planar pouch motors make other alternative offering a scalable and printable approach to fluid‐driven thickness morphing. Here the example include the bonded‐sheet pouch actuators comprising of linear and rotary units: a three‐pouch linear actuator produced ∼28% stroke and ∼100 N tensile force at 40 kPa, while a hinged rotary pouch generated ∼0.2 N·m torque at 20 kPa [152]. Extending this concept further, fluid‐driven origami muscles demonstrated extreme volumetric morphing, achieving ∼90% one‐dimensional (1D) contraction, ∼92% 2D area contraction, and ∼91% 3D volume reduction. Demonstrations included lifting a 22 kg car wheel, underscoring the exceptional force‐to‐weight ratio achievable in soft, fluid‐filled shells [87].

For tactile interfaces and high‐density haptic displays, microfluidic and pneumatic arrays have been developed to translate pressure into fingertip‐scale doming. Here the example include a stacked microfluidic pneumatic array, which achieved an out‐of‐plane height of 0.145 mm and a force of 17.7 mN per pixel at 1000 mbar, with a spatial resolution of 1.25 mm [153]. These results demonstrate precise, repeatable vertical deformation suitable for dense haptic feedback systems. Fluid‐based actuation has also been combined with alternative driving mechanisms and materials. For instance, a dielectric fluid‐based haptic actuator consisted of a thin oil‐filled pouch with a 1.5 mm diameter opening covered by a silicone membrane [154]. The device achieved a bump height of 1.45 mm at 3 kV and a force of 13 mN at 3.5 kV using approximately 10 µL of oil, with perceivable vibration at 200 Hz. Pneumatic actuation principles have likewise been implemented using soft lithography [155]. An array of up to 36 pneumatic out‐of‐plane actuators, fabricated by molding PDMS using dual SU‐8 mold halves, is one example [156]. The actuators exhibited maximum bending angles of ∼30° and horizontal tip displacements of ∼220 µm at a supply pressure of 1 bar. Hybrid electro‐pneumatic systems further enhance fluid‐driven thickness modulation. This is reflected through the tactile actuators that combine pneumatic pressure and electrostatic actuation in a circular elastomer membrane (3 mm diameter, 0.1 mm thick) with compliant electrodes and pneumatic valves [157]. Applying 10 kPa air pressure and 472 V generated a 0.5 mm‐high protrusion capable of stimulating human skin. Reducing the voltage led to pressure drop and membrane retraction, enabling controllable, reversible out‐of‐plane motion.

Collectively, these studies demonstrate that fluid‐driven actuators by tailoring chamber geometry, skin stiffness, reinforcement strategy, and working pressure (positive or negative) can deliver millimeter‐to‐centimeter‐scale reversible out‐of‐plane shrinkage and expansion with application‐relevant forces and bandwidths. While challenges remain in terms of system integration, response speed, and portability, fluid‐driven architectures remain among the most powerful and versatile technologies for thickness‐modulating soft skins, particularly in haptic and morphing interface applications.

### 3.4 Thermal/Shape Memory Actuation

Thermally‐driven and shape memory–based actuators convert heat into mechanical work through phase transitions, molecular reconfiguration, or temperature‐dependent modulus changes, which can be architected to generate large, reversible out‐of‐plane deformation. In unstructured or constrained environments, these actuators are particularly attractive because they can be compactly embedded, electrically powered through simple wiring, and programmed to pop out‐of‐plane to grasp, lift, or reconfigure surfaces before retracting upon cooling. Their robustness and material‐level programmability make them well suited for deployable soft skins and morphing interfaces.

Shape memory alloy (SMA) actuators, particularly those based on Joule‐heated NiTi wires, provide exceptionally high‐power density contraction. When routed as tendons or embedded within compliant matrices, SMA contraction can be mechanically amplified to produce large out‐of‐plane bending and doming. Using free‐sliding SMA tendons decoupled from the bulk silicone body length, the out‐of‐plane bending angles up to 400° have been demonstrated with tip forces of approximately 0.89 N, enabling a soft gripper capable of lifting objects up to 1.5 kg [158]. Casting SMA elements into curved smart soft composites further increases mechanical advantage, as demonstrated by a curved SMA bending element that delivered nearly 3 times the lifting force of an equivalent straight design during soft gripper operation [159]. Textile‐reinforced SMA soft actuators have also been reported, integrating strain sensing and achieving bending angles of up to 270° at an input power of 18 W, offering compact out‐of‐plane strokes with built‐in proprioceptive feedback [160]. Together, these studies show that SMA contraction can be effectively routed or amplified to realize reversible shrink–expand motion normal to the surface while maintaining simple electrical drive schemes.

Shape memory polymers (SMPs) provide an alternative thermal actuation route based on temperature‐triggered softening and strain recovery. Thermally activated SMP–elastomer laminates can be programmed to deploy from flat to 3D geometries. One example is the direct 3D‐printed SMP–elastomer bilayer that is heated through the SMP glass transition temperature, releasing stored compressive strain and causing star‐shaped sheets to rise into domes and lattices to expand out‐of‐plane [161]. Curvature saturated near ∼60°C, and printed lattices exhibited approximately 62% compaction change during thermal cycling. These thin, monolithic structures demonstrated reliable and repeatable out‐of‐plane expansion upon heating, followed by shrinkage during cooling or reprogramming, with deformation geometry governed by print pattern and layer sequencing.

Thermally responsive liquid crystal elastomers (LCEs), discussed in more detail in Section 4.1, also fall within the broader class of thermal actuators. LCEs contract along their director and expand transversely when heated. By patterning radial +1 defects and laminating multiple aligned films, it is possible to form conical domes upon heating, achieving peak heights of ∼3.4 mm, approximately 70 times the film thickness [162]. These actuators sustained loads exceeding 1100 times their own weight and achieved specific work values near 19 J kg^−^^1^, while maintaining stroke under positive pressures up to 7 kPa [162]. Such architectures demonstrate high‐stroke, thermally‐driven out‐of‐plane lifters that reversibly shrink and expand under modest thermal inputs and can be tiled into arrays to create programmable surface topographies.

Phase‐change composites provide another compact approach to thermal out‐of‐plane actuation. Paraffin–PDMS composites exploit the ∼10%–20% volumetric expansion of wax upon melting to bulge a compliant membrane normal to the surface. Here, an example is the micromachined actuator incorporating a polyimide–aluminum microheater, which produced reversible vertical strokes up to 160 µm over a 1 mm diameter active area at approximately 160 mW input power [163]. This corresponded to an ∼18% composite volume change between 20°C and 80°C and enabled lifting of 60 mg test masses. Arrays of such cells can be integrated to form programmable surface bumps that expand upon heating and shrink upon cooling using simple embedded heaters.

From a system‐level perspective, each thermal and shape memory technology presents distinct trade‐offs. SMA tendons offer the largest out‐of‐plane rotation per footprint and straightforward electrical integration but are limited by cooling‐dominated cycle rates and thermal hysteresis, which can be mitigated through textile integration or active cooling strategies. SMP and LCE sheets enable scalable, large‐area strokes with excellent shape programmability and moderate force output; localized resistive traces or thin‐film heaters help minimize thermal mass and improve response. Paraffin‐based composites provide compact, high‐energy‐density doming suitable for board‐level or microscale features, with cycle time governed primarily by heater design and thermal management.

Collectively, thermal and shape memory actuators satisfy the core requirement of reversible out‐of‐plane shrinkage and expansion and remain a versatile class of technologies for soft skins. Their selection depends on the desired balance among stroke amplitude, load capacity, bandwidth, energy efficiency, and integration constraints within morphing and haptic interface systems.

### 3.5 Light‐Driven Actuators

Light provides a powerful stimulus for remote, rapid, and spatially selective control of shrinkable and expandable out‐of‐plane soft actuators. Unlike electrically or fluidics‐driven systems, light‐driven actuation enables wireless operation, high spatial addressability, and access to confined or sealed environments where physical connections are impractical. Two dominant light‐based transduction routes had been reported in literature: first, photochemical actuation relies on azobenzene or related chromophores, where photoisomerization reduces nematic order under illumination, generating contractile strain along the local director field. When spatially programmed, this mechanism produces out‐of‐plane deformation modes such as doming, coning, and thickness undulation [164]. Second, photothermal actuation incorporates light absorbers that convert optical energy into heat, driving nematic‐to‐isotropic transitions in LCEs or volume phase transitions in hydrogels, which in turn generate curvature, doming, or global contraction normal to the surface [165, 166]. LCEs and liquid crystal networks (LCNs) are particularly well suited to light‐driven out‐of‐plane morphing due to their programmable anisotropy. By encoding spatially varying director fields, localized illumination can generate through‐thickness strain gradients that translate in‐plane contraction into vertical expansion or shrinkage. A representative photothermal design integrates an LCE with a thin MXene‐based sensing layer to form a bimorph membrane that bends and pops out‐of‐plane under near‐infrared illumination. The membrane exhibits tensile strength of approximately 16.3 MPa, light‐driven actuation stress near 1.56 MPa, and integrated strain sensing with a gauge factor of about 4.72, enabling closed‐loop control of out‐of‐plane strokes and flapping motions under laser excitation [165].

Photochemical LCNs enable genuinely surface‐normal deformation without bulk heating. In polydomain coatings, ultraviolet illumination induces reversible surface relief characterized by jagged spikes with amplitudes exceeding 20% of the film thickness, which erase within approximately ten seconds after the light is removed [164]. These results demonstrate both large reversible thickness modulation at the surface and substantial out‐of‐plane bending in free‐standing films, driven purely by light and programmable through molecular alignment.

Beyond static deformation, light can also sustain cyclic out‐of‐plane shrinkage and expansion. In LCN cantilevers, continuous illumination has been shown to induce self‐oscillatory behavior. Bending oscillations reach frequencies near 80 Hz, while contraction–expansion cycles occur at approximately 0.5–18 Hz depending on geometry and beam placement, all without physical tethers or wiring [167]. Such light‐driven oscillations are directly relevant for cyclic pumping, fluttering, and active ventilation in constrained environments.

Photothermal actuation has also been extensively explored in hydrogel‐based systems. Near‐infrared‐responsive hydrogels loaded with graphene oxide, gold nanorods, or similar absorbers undergo rapid volume shrinkage above their lower critical solution temperature and recover upon cooling. When laminated to an elastomer layer, the resulting thermal strain mismatch produces pronounced out‐of‐plane curvature and doming. For example, a PNIPAM–graphene oxide–HEMA hydrogel bonded to PDMS, discussed in Section 3.5, achieved a bending angle change of approximately 342° within about 90 s under simulated sunlight at an intensity of ∼1.2 W cm^−^^2^. Optimal performance was observed for a hydrogel thickness of approximately 0.5 mm and a graphene oxide concentration near 2 mg mL^−^^1^ [166]. Similar architectures can be actuated using near‐infrared lasers to achieve localized strokes, gripping, or surface protrusions, making them suitable for out‐of‐plane actuation in cluttered or confined environments where line‐of‐sight access is available.

For effective light‐driven out‐of‐plane shrinkage and expansion, three design considerations dominate performance. First, absorber selection and wavelength choice must balance penetration depth and heating rate. Near‐infrared wavelengths minimize parasitic absorption by polymers and water, improving actuation uniformity in thicker films [165, 166]. Second, director field programming should place contractile axes through the thickness so that illumination generates controlled strain gradients corresponding to the desired out‐of‐plane mode. Third, thermal management is critical: thin active layers and high interfacial thermal conductance accelerate response while limiting heat accumulation and protecting surrounding materials. With these design strategies, light‐driven actuators offer wire‐free, addressable, and repeatable out‐of‐plane deformation, complementing electrically and fluidically‐driven approaches. Their unique combination of spatial selectivity and remote operation makes them particularly attractive for adaptive soft skins, haptic interfaces, and morphing surfaces operating in unstructured and access‐limited environments.

While the actuation stimuli discussed in Section 3 defines how external energy is supplied and transduced into mechanical work, they do not alone determine the achievable magnitude, reversibility, or stability of out‐of‐plane deformation. These performance characteristics are ultimately governed by the material platform in which actuation is embedded, including its intrinsic anisotropy, phase behavior, transport properties, and mechanical compliance. Accordingly, the following section shifts focus from stimulus‐centric mechanisms to material‐centric strategies, examining how specific classes of soft materials encode and regulate reversible thickness morphing across a wide range of actuation modalities.

Overall, the out‐of‐plane morphing strategies discussed in this section utilize distinct deformation principles and exhibit different performance characteristics and application suitability. The relative advantages and limitations of these approaches in terms of stroke, force output, response speed, power demand, and scalability are compared comprehensively in Table 2, highlighting their complementary roles and the growing importance of hybrid design strategies for next‐generation morphing skins.

**TABLE 2:** Comparison of representative approaches for reversible out‐of‐plane thickness morphing in soft skins.

Actuation stimulus	Material platform	Rep. architectures	Stroke	Force	Bandwidth	Scalability and integration	Key advantages	Key limitations	Rep. Refs
Electrostatic	Dielectric elastomers	Doming membranes, multilayer DEAs	0.1–2 mm	mN–N	up to 10^2^ Hz	Moderate; arrays feasible	Fast response, compact, silent	High voltage (kV), dielectric breakdown, limited force	[125, 230]
	Electrohydraulic liquids	Zipping cells, pouches, HAXEL taxels	0.1–2.5 mm	0.1–2 N	DC–200 Hz	High; dense arrays demonstrated	Large stroke at low mass, good force density	High voltage (kV), sealing, charge retention	[130, 210, 231]
Electromagnetic	Coil–magnet elastomers	Attraction/repulsion diaphragms	0.2–5 mm	0.1–1 N	1–200 Hz	Moderate; module‐level integration	Low voltage, bidirectional, strong force	Joule heating, EMI, added mass	[137, 140]
	Liquid‐metal coils	Soft embedded electromagnetic skins	0.5–3 mm	mN–N	up to 100 Hz	Moderate; flexible routing	Extreme compliance, reconfigurable	Heating, fabrication complexity	[26, 111]
Fluid‐driven	Pneumatic elastomers	Microchambers, pouch motors	mm–cm	N–10^2^ N	<10 Hz	High; large‐area skins	Large force and stroke, simple materials	Tethers, slow dynamics, bulky systems	[232]
	Vacuum‐driven structures	Origami and buckling actuators	cm‐scale	10^2^ N	Low	Moderate	High contraction ratios, strong actuation	Vacuum source, sealing	[150, 151, 233]
Thermal/shape‐memory	SMAs	Tendon‐driven or biasing layers	mm–cm	High	<10 Hz	Moderate	Very high force density, simple wiring	Slow cooling, hysteresis, fatigue	[158, 159, 234, 235]
	SMPs	Bilayers, lattices, morphing shells	0.1–10 mm	Low–moderate	Very low	High via printing	Shape locking, easy fabrication	Slow response, thermal inefficiency	[18]
	LCEs (thermal)	Director‐programmed domes and cones	mm‐scale	mN–N	Low–moderate	High via printing	Programmable morphologies, large strain	Thermal bottleneck, control complexity	[79, 86]
Light‐driven	Photo‐LCEs	Optical doming, Braille displays	0.1–2 mm	mN	Low	Moderate	Wireless, spatially selective	Line‐of‐sight, slow relaxation	[176, 177]
	Photothermal composites	Local heating‐induced bulging	0.1–5 mm	Low–moderate	Low	Moderate	Simple stimulus delivery	Thermal diffusion, efficiency	[166, 185]
	Photo‐responsive hydrogels	Swelling‐based thickness change	mm–cm	Low	Very low	Low–moderate	Soft, biocompatible	Slow response, dehydration	[236, 237]

## 4 Mechanics and Material Platforms Enabling Reversible Thickness Morphing

Beyond the choice of actuation stimulus, the ability of a soft skin to reversibly expand and contract in thickness is fundamentally governed by the material platform in which deformation is encoded. A wide spectrum of materials has been explored to enable conformable systems with biomimetic tactile response and haptics capability using thickness‐modulating, including soft matter systems such as liquids, gels, colloids, polymers, foams, and biological tissues [31, 168]; organic materials [119]; nanomaterials such as graphene, nanowires, and CNTs [169]; and composite systems incorporating liquid metal alloys or rheological fluids [170]. Among these, soft polymeric and elastomeric materials have been most extensively investigated due to their intrinsic mechanical compliance and their ability to closely match the modulus of human skin. Materials with skin‐like compliance not only improve wearer comfort but also enhance the fidelity of mechanical signal transmission, making them particularly attractive for soft skins designed to deliver perceptible out‐of‐plane haptic feedback [118]. Importantly, many material platforms, including LCEs, hydrogels, shape memory polymers, and biohybrid tissues can support multiple actuation stimuli, with their response governed by internal structure, anisotropy, or phase behavior rather than the external drive alone.

This section therefore focuses on material‐centric platforms for reversible thickness morphing, highlighting how intrinsic material properties, microstructure, and architectural programming enable doming, buckling, swelling, or contraction normal to the surface. Emphasis is placed on deformation magnitude, reversibility, response speed, durability, and compatibility with skin‐like interfaces, providing a material‐level perspective that complements the stimulus‐driven discussion in Section 3.

### 4.1 Liquid Crystal Elastomers (LCEs)

Liquid crystal elastomers (LCEs) constitute a distinctive class of soft active materials that convert programmed in‐plane contraction into large, reversible out‐of‐plane shape change, enabling both shrinkable depressions and expandable protrusions in thin films and sheets. Owing to this capability, LCEs have attracted growing interest for applications in soft robotics, micromachines, artificial muscles, tissue engineering, and adaptive interfaces [171]. Structurally, LCEs are anisotropic polymer networks that combine the elasticity of rubber with the orientational order of liquid crystals, resulting in exceptional actuation, optical, and mechanical properties. A defining feature of LCEs is their strong response to thermal stimulation, which induces reversible changes in molecular alignment within the polymer network and drives macroscopic deformation [172, 173]. Depending on molecular alignment and architectural programming, LCEs can contract, elongate, bend, twist, or buckle [174]. Beyond thermal actuation, LCEs can also be stimulated using light, magnetic fields, or electrical inputs, broadening their applicability across different operational contexts. In all cases, actuation arises from the release of stored elastic energy when the external stimulus is applied and subsequently removed, enabling cyclic and reversible motion [171]. Importantly, by carefully designing the stimulus distribution and alignment patterns, researchers have demonstrated the ability to program complex shapes, deformation pathways, and shape memory effects into LCE‐based systems [175].

Hybrid material strategies have further extended LCE functionality. For example in [176] an optically actuated soft actuator combining LCEs with CNTs was developed for Braille display applications. Under laser irradiation with optical power up to ∼60 mW, the actuator produced contractions of approximately 40 µm with stabilization times below 6 s, demonstrating localized and addressable out‐of‐plane deformation. Such composite approaches improve photothermal efficiency while retaining the intrinsic softness and reversibility of LCEs.

Additive manufacturing has played a transformative role in enabling spatially programmed LCE architectures [86]. For example, high‐operating‐temperature direct ink writing (HOT‐DIW)‐based LCE actuators allow precise alignment of mesogen domains along the printing path, as well as arbitrary form factors and programmable deformation modes (Figure 6a). Similarly, shape‐switching LCEs functionalized with supramolecular crosslinks, dynamic covalent bonds, and azobenzene groups were employed in 4D printing to realize Braille‐like actuator arrays (Figure 6b) [177]. The refreshable tactile device have also been developed by harnessing opto‐mechanical stress gradients generated under illumination [178]. These devices enable controlled force generation and surface reconfiguration [179]. These approaches support scalable fabrication of large actuator arrays with individually programmable out‐of‐plane responses.

**FIGURE 6:**
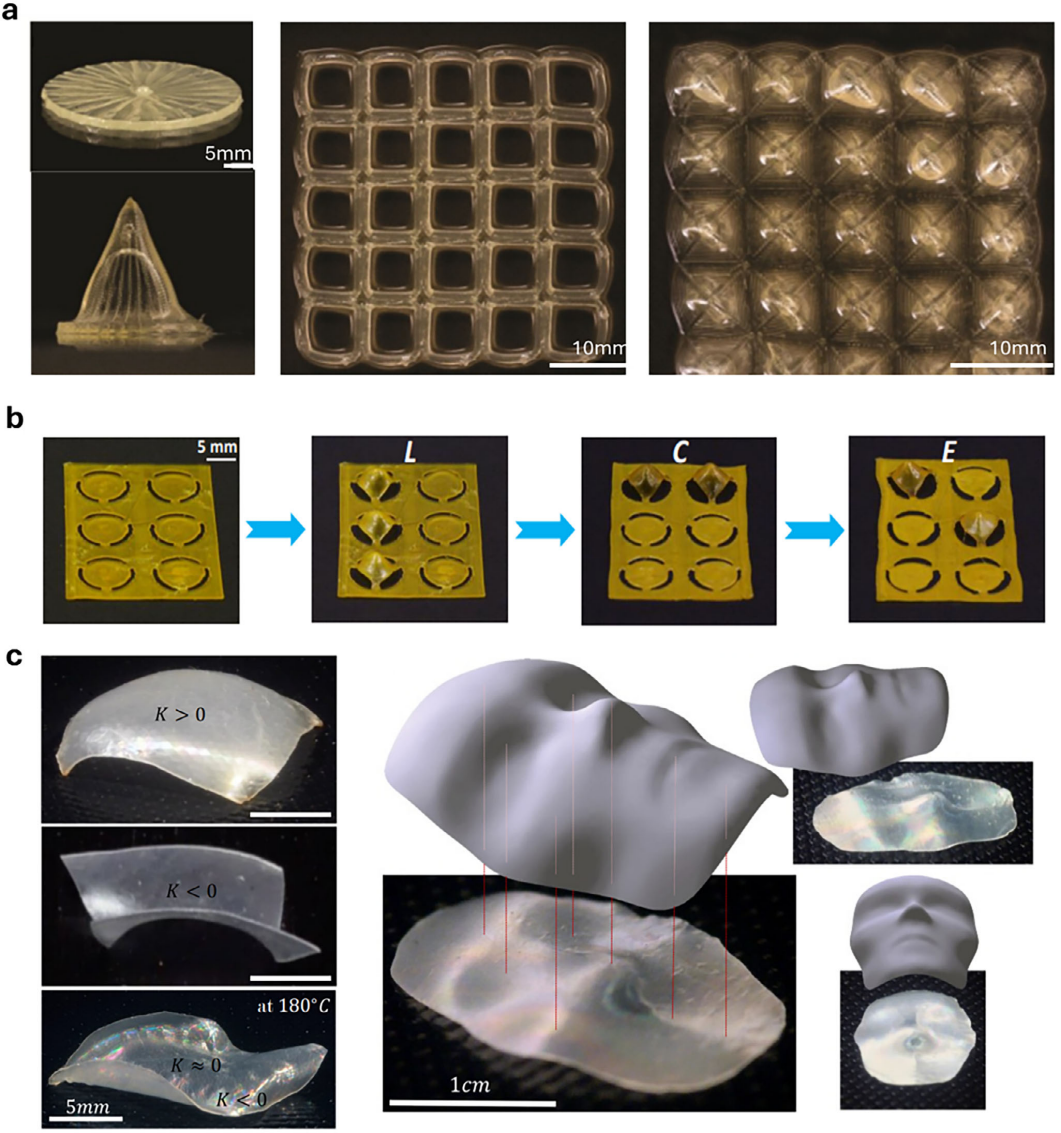
LCE‐based reversible thickness morphing actuators. (a) Arrayed LCE microstructures demonstrating scalable thickness modulation across large areas, highlighting uniform and repeatable deformation suitable for tactile and surface morphing applications. Adapted with permission [86]. Copyright 2018, Wiley. (b) LCE‐based Braille actuator concept showing programmable, light induced height modulation of discrete surface features, with reversible switching between raised and flattened states over repeated activation cycles. Adapted with permission [177]. Copyright 2020, Wiley. (c) Programmable LCE sheets exhibiting complex 3D shape morphing through spatially patterned nematic director fields, enabling reversible transitions between flat and curved geometries. Adapted from Ref. [79] under the PNAS license for noncommercial and educational use. Copyright 2018, National Academy of Sciences.

Beyond discrete actuators, patterned LCE films enable deterministic surface morphing. Photopatterned LCE coatings can generate surface depressions from splay alignment and elevations from bend alignment, producing thermally‐driven topographies that reversibly rise and subside on demand [180]. Building on this mechanism, laminated LCE sheets incorporating arrays of +1 topological defects form conical domes that lift heavy loads while preserving millimeter‐scale stroke, achieving specific work values approaching 20 J kg^−^^1^ with peak heights on the order of millimeters [162]. More generally, inverse design rules now allow the programming of arbitrarily curved surfaces such that thin nematic elastomer sheets morph into prescribed 3D shapes upon heating and relax upon cooling (Figure 6c) [79]. Additive manufacturing further extends this concept to printed beams and lattices that bend and twist out‐of‐plane with large, reversible strains [86].

Electrical driving of LCEs has also been explored to improve response speed and integration. For example, monolithic dielectric LCE actuators coupled shape programmability with efficient electrical stimulation can produce buckling and doming motions to lift loads hundreds of times their own weight [181]. At the extreme end of dynamic performance, LCE sheets encoded with topological patterns and designed for snap‐through instabilities have demonstrated rapid transitions between flat and curved states, enabling explosive out‐of‐plane motion and even leaping behavior, highlighting the upper limits of speed and power density achievable in LCE‐based systems [182].

Despite several advantages, LCE actuators face inherent challenges related to low thermal conductivity (∼0.3 W m^−^^1^ K^−^^1^) and slow passive cooling, which is often 5 to 50 times slower than heating. These limitations restrict cycle rate and complicate closed‐loop control [183]. To overcome these constraints while enabling shrinkable out‐of‐plane actuation, researchers have increasingly relied on programmed nematic order to transform large thermotropic contraction into doming, coning, and spiraling motions. For example, 3D‐printed monodomain LCEs exhibit contractions of ∼43.6% along the director with ∼29.8% transverse expansion; patterned sheets can pop into cones reaching heights of ∼6.5 mm with out‐of‐plane stroke ratios up to ∼1628%, delivering ∼39 J kg^−^^1^ and lifting loads up to 1000 times their own weight [86].

Advanced chemistries further enhance performance and reusability. Siloxane‐based LCEs with switchable thermal reprogrammability achieve actuation strains up to ∼52.4% and can be remolded into 3D convex actuators whose tops rise and fall reversibly under heating and cooling, while lifting loads approximately 10 times their own mass [184]. Vitrimer LCEs introduce photothermal drive alongside self‐weldable and recyclable interfaces: under 365 nm illumination at 160 mW cm^−^^2^, these materials bend and bloom out‐of‐plane with near‐ideal shape fixing (R_f_ ≈ 98.6%) and recovery (R_r_ ≈ 99.8%), lifting loads up to ∼7:1 relative to actuator mass [185]. To address thermal bottlenecks directly, integrating soft thermoelectric layers between pre‐strained LCE sheets enables active cooling and bidirectional out‐of‐plane bending. Such systems achieve average blocking forces of 0.138 N over 98 cycles, peak forces of 0.35 N, and reduce cooldown time to approximately 43% of heating time, supporting higher duty‐cycle operation for thickness‐morphing tasks [183].

Collectively, these advances position LCEs as a uniquely powerful material platform for reversible thickness morphing, offering programmable geometry, large strain, and high work density in thin, compliant skins. While challenges in thermal management and response speed remain, continued progress in material chemistry, alignment programming, and hybrid integration is rapidly expanding the applicability of LCEs for haptic interfaces, adaptive surfaces, and morphing soft skins operating in complex environments.

### 4.2 Hydrogels and Osmotic Systems

Hydrogels and osmotic actuators convert solvent transport, phase transitions, and osmotic pressure gradients into mechanical work that can be patterned to generate large, reversible out‐of‐plane deformation. Their high compliance, aqueous compatibility, and intrinsic softness make them attractive candidates for adaptive skins, haptic interfaces, and biointegrated systems. Two complementary actuation strategies dominate this class of materials. First, swelling mismatch in thin bilayers or patterned sheets produces curvature, doming, or plate bending that expands and shrinks normal to the surface in response to thermal, optical, or chemical stimuli [166, 186]. Second, osmotic pressure–driven actuation, achieved through membranes or controlled solute concentration gradients, enables strong out‐of‐plane bulging and recovery using low input power and simple aqueous chemistry [187, 188, 189].

Thermoresponsive hydrogels based on poly(N‐isopropylacrylamide) (PNIPAM) exhibit lower critical solution temperature (LCST) behavior: they deswell or shrink above the transition temperature and swell below it. When incorporated into asymmetric bilayer architectures, this volumetric change generates a through‐thickness strain gradient that induces out‐of‐plane curvature. For example, PNIPAM/graphene oxide bilayers demonstrate rapid, reversible, and bidirectional bending under either thermal or near‐infrared stimulation, with response times on the order of 1 min. The direction and magnitude of bending can be precisely programmed through layer composition and thickness [186]. Similarly, composite PNIPAM–graphene oxide–hydroxyethyl methacrylate hydrogels laminated to polydimethylsiloxane (PDMS) exhibit large out‐of‐plane bending when exposed to hot solutions, simulated sunlight, or laser illumination. The enhanced response speed in these systems is attributed to improved water transport and efficient photothermal conversion within the hydrogel layer [166]. Collectively, such bilayer designs enable wireless, addressable, and reversible out‐of‐plane expansion and shrinkage in thin, skin‐like form factors.

Beyond thermally‐driven swelling, osmotic actuation offers a powerful mechanism for generating large vertical strokes by confining hydrogels behind selectively permeable membranes that sustain substantial osmotic pressure differences. In one example, a turgor‐based osmotic actuator generated actuation stresses of approximately 0.73 MPa over ∼96 min using 1.16 cm^3^ of hydrogel. Incorporating electroosmotic transport to accelerate solvent flow increased both speed and stress, achieving ∼0.79 MPa in roughly 9 min [187]. In this architecture, osmotic pressure drives doming of a compliant boundary normal to the surface, demonstrating strong and repeatable out‐of‐plane expansion and contraction.

More compact osmotic systems have been realized using forward osmosis. In one example, the forward‐osmosis‐driven actuator drew water across a membrane into an actuation chamber with a compliant wall, achieving characteristic response times of 2–5 min for a 10 mm‐scale device while producing forces exceeding 20 N at power levels on the order of milliwatts [188]. Mechanical work is transduced through bulging of the compliant wall, providing an efficient route to out‐of‐plane displacement with minimal electrical burden.

Reversible control of osmotic pressure can also be achieved through electrosorption, in which osmolyte concentration is modulated using flexible porous carbon electrodes at low voltages (∼1.3 V) [189]. A tendril‐like soft robot based on this principle demonstrated reversible stiffness modulation of approximately fivefold and large bending rotations while operating in aqueous environments. Although demonstrated in a slender geometry, the same concentration‐control strategy can be extended to membranes and sheet‐like architectures to realize cyclic out‐of‐plane shrink–expand motion using safe voltages and simple materials.

From a design perspective, maximizing out‐of‐plane stroke and response speed in hydrogel and osmotic actuators requires careful control of geometry and transport pathways. Thin active layers reduce poroelastic time constants, while semipermeable or low‐permeability interfaces help localize osmotic pressure where bulging or doming is desired. Photothermal fillers or Joule‐heated microheaters can bias thermoresponsive hydrogels without external pumps, whereas electrosorption modules enable reversible osmolyte control for closed‐loop osmotic actuation. Membranes must retain selectivity under repeated cycling and mechanical load, and interfaces should be engineered for high thermal and hydraulic conductance to accelerate recovery.

With appropriate material selection and architectural design, hydrogel and osmotic actuators provide scalable, wire‐free, and repeatable out‐of‐plane shrinkage and expansion. Although their response times are typically slower than those of electrostatic or electromagnetic systems, their low power requirements, intrinsic softness, and compatibility with aqueous and biological environments make them particularly well suited for adaptive soft skins operating in unstructured or access‐limited settings.

### 4.3 Shape Memory Polymers and Composites

Shape memory polymers (SMPs) and SMP‐based composites constitute an important class of material platforms for reversible thickness morphing due to their ability to store and release large, programmed strains through thermally activated phase transitions (Figure 7). Unlike elastomeric actuators that require continuous stimulus input to sustain deformation, SMPs exploit a temporary shape fixed below a characteristic transition temperature typically the glass transition temperature (T_g_) and recover their programmed geometry upon heating. This property enables soft skins that remain compact or flat during standby and expand or protrude out‐of‐plane only when activated, making SMPs particularly attractive for deploy‐on‐demand interfaces.

**FIGURE 7:**
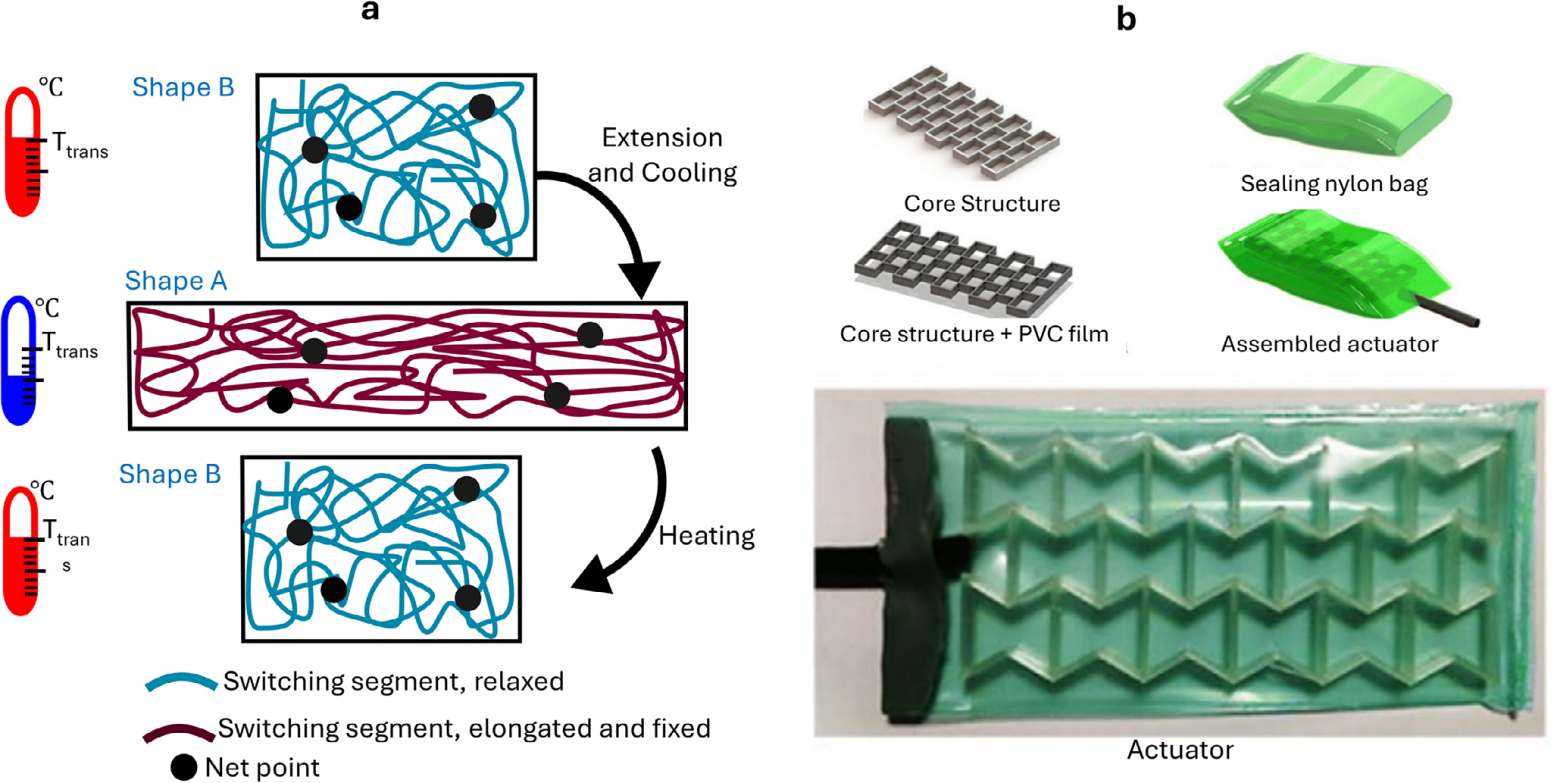
(a) Shape memory polymers. (b) Shape memory alloy‐based actuator (a) and (b). Adapted under the terms of the Creative Commons Attribution 4.0 International License [119]. Copyright 2023, The Author(s).

For thickness‐modulating soft skins, SMPs are most commonly implemented in bilayer or multilayer architectures, where recovery of pre‐strain in the SMP layer induces curvature, doming, or buckling normal to the surface. Additive manufacturing and 4D printing approaches have further expanded the design space, allowing spatial programming of recovery strain and stiffness to generate complex 3D morphologies from initially planar sheets [161]. Such approaches enable large out‐of‐plane deformation while maintaining a low device profile and minimal actuation hardware.

SMP composites combine shape memory behavior with enhanced mechanical robustness or multifunctionality by incorporating elastomers, fibers, or conductive fillers. These hybrid designs mitigate some inherent limitations of SMPs such as low actuation stress and slow thermal response while preserving excellent shape programmability [119, 190]. Compared to hydrogels or electroactive polymers, SMP‐based actuators typically exhibit superior mechanical durability and resistance to environmental degradation, although this often comes at the expense of bandwidth and continuous reversibility [190].

Overall, SMPs and SMP composites are best suited for applications where large, reversible out‐of‐plane deployment is required but high actuation speed is not critical, such as reconfigurable haptic features, adaptive protective skins, and morphing surfaces. Their compatibility with scalable manufacturing and architected designs positions them as an important complementary platform within the broader ecosystem of thickness‐morphing soft skins.

### 4.4 Biohybrid and Living Materials

Biohybrid actuators harness the contractile work of living muscle cells to generate mechanical strokes that reversibly shrink and expand structures out‐of‐plane. In these systems, aligned cardiomyocytes or skeletal muscle cells are laminated onto compliant elastomeric substrates or patterned onto microfabricated skeletons, such that each excitation cycle produces a rapid active contraction followed by passive elastic recovery. Because the active contractile layer is thin, compliant, and spatially addressable, biohybrid actuators can generate large curvature, doming, or bending normal to the surface while preserving softness, adaptability, and safety key attributes for operation in unstructured and constrained environments.

A noteworthy example is the medusoid biohybrid actuator [191], which reproduces the bell contraction of a jellyfish using dissociated rat cardiomyocytes patterned onto a silicone elastomer. Electrical field pacing synchronizes contraction across the muscle sheet, causing the compliant bell to contract out‐of‐plane and subsequently relax, producing thrust through repeated shrink–expand cycles. The system was designed by matching structural layout, stroke kinematics, and fluid–structure interactions observed in biological jellyfish, establishing a foundational design framework for thin biohybrid membranes that repeatedly deform normal to the surface with high fidelity [191].

Biohybrid architectures capable of more complex and addressable out‐of‐plane motion have also been demonstrated. A tissue‐engineered ray was realized by patterning a single layer of cardiomyocytes into serpentine circuits on an elastomeric body reinforced with an asymmetric gold skeleton [192]. Periodic excitation produced undulatory fin strokes that deflected out‐of‐plane, generating propulsion. Direction and speed were controlled using light pacing at frequencies between one and three hertz. The device maintained stable cruising over distances exceeding 99.5 mm with more than 80 stroke cycles and achieved a maximum linear speed of approximately 3.2 mm s^−^^1^ at near two‐hertz pacing. Measured out‐of‐plane fin deflections closely matched those of live rays, demonstrating biofidelity and precise, reversible bending without mechanical tethers. Such features are advantageous for navigating cluttered environments where line‐of‐sight optical control is feasible.

Long‐term durability and autonomous operation have also been demonstrated using a biohybrid fish driven by two opposing cardiac muscle layers [193]. The antagonistic muscle layers were separated by an insulating node that enabled intrinsic pacing and bidirectional electromechanical coupling. This configuration generated cyclic up–down tail strokes that bent out‐of‐plane and propelled the body forward. Performance improved during the first month of culture and subsequently stabilized, with spontaneous swimming and optogenetic controllability maintained for at least 108 days corresponding to approximately 38 million contraction cycles [193]. These results highlight the remarkable endurance of living muscle when integrated with compliant skeletons and built‐in feedback pathways, enabling sustained, reversible out‐of‐plane shrink–expand motion.

Across biohybrid systems, the achievable out‐of‐plane stroke amplitude and performance are governed by three primary design choices. First, muscle alignment and patterning define the principal contraction axis through the thickness and along the deforming element, setting the direction and magnitude of out‐of‐plane motion. Second, the passive elastic skeleton stores strain energy during contraction and releases it during recovery, enhancing cycle symmetry and reducing the energetic burden on the muscle layer [192]. Third, the excitation method and frequency determine the balance between contraction speed and fluid refill or elastic recovery, with pacing near two hertz maximizing cruise speed in ray‐inspired architectures, while lower frequencies favor efficient refill in jellyfish‐like pumping systems [191, 192].

While most reported biohybrid systems emphasize distributed bending or traveling‐wave deformation across an entire structure, the underlying actuation principles are directly extensible to localized thickness modulation and out‐of‐plane expansion–shrinkage in soft skins. By confining aligned muscle layers within laminated elastomeric stacks, pocketed chambers, or dome‐shaped membranes, the intrinsic contractile strain of living cells can be redirected along the surface‐normal direction to produce controlled vertical protrusion and retraction. For example, antagonistic muscle layers arranged above and below a compliant cavity could generate bidirectional thickness change, analogous to hydrostatic or electrostatic doming mechanisms discussed in earlier sections. Similarly, patterning cardiomyocytes around circular or radial geometries would enable synchronized contraction toward a central axis, yielding localized out‐of‐plane bulging suitable for haptic or morphing interfaces. Importantly, the passive elastic skeleton in biohybrid systems already serves as an energy storage and recovery element; redesigning this skeleton to bias deformation through thickness rather than along the surface provides a clear pathway to shrinkable and expandable biohybrid skins that are also wire‐free. Such architectures would uniquely combine self‐powered actuation, inherent compliance, distributed sensing, self‐repair at the material level, and long‐term cyclic durability, positioning biohybrid materials as a promising though still exploratory route toward adaptive, living soft skins capable of autonomous thickness morphing in unstructured environments. The challenges remain in terms of environmental robustness, nutrient supply, and long‐term integration.

The material platforms described in Section 4 provide the foundational mechanisms by which thickness morphing can be programmed, amplified, or stabilized within soft skins. However, translating these capabilities into practical haptic interfaces and deployable systems requires integration beyond the material level. Challenges such as sensing co‐location, scalable fabrication, power delivery, durability under cyclic loading, and real time control must be addressed at the system architecture level. The next section therefore focuses on integrated and hybrid designs that combine actuation, sensing, and control into functional thickness‐morphing soft skins suitable for real‐world operation.

### 4.5 Mechanics of Thickness Morphing

Thickness morphing in soft skins is fundamentally governed by mechanics, as surface‐normal displacement emerges from the redistribution of incompatible in‐plane strains within compliant structures. While actuation mechanisms such as electromagnetic, electrostatic, thermal, or fluidic inputs provide the driving stimulus, they may not be directly producing out‐of‐plane deformation. Instead, they induce spatially non‐uniform strains that cannot be accommodated within a planar configuration, leading to curvature and thickness change. This perspective is consistent with the mechanics of thin plates and shells with incompatible strains, as described in non‐euclidean elasticity [194, 195].

For a thin active sheet, the in‐plane strain can be expressed using the Föppl–von Kármán [196] framework as:

(1) $$\varepsilon_{\alpha \beta }=\frac{1}{2}\;\left(u_{\alpha ,\beta }+u_{\beta ,\alpha }+w_{,\alpha }w{,}_{\beta }\right)-\varepsilon_{\alpha \beta }^{*}$$

where  *u*_α_ denotes in‐plane displacement, *w* is the surface‐normal displacement, and $\varepsilon_{\alpha \beta }^{*}$ represents actuation‐induced or residual strain. The associated curvature tensor is given by:

(2) $$\kappa_{\alpha \beta }=-\;w_{,\alpha \beta }$$

These relations establish the direct coupling between in‐plane strain and out‐of‐plane deformation. When the imposed strain field $\varepsilon_{\alpha \beta }^{*}$ is incompatible with a flat geometry, the structure relaxes through bending or buckling, offering the possibility of thickness modulation [196]. The transition from in‐plane strain to surface‐normal deformation is governed by the competition between stretching and bending energies. The elastic energy (*U*_s_) and bending energy (*U*_b_) of a thin structure can be expressed as:

(3) $$U_{s}∼Y\int_{A}\varepsilon^{2}\;dA$$

(4) $$U_{b}∼B\int_{A}\kappa^{2}\;dA$$

where ε is in‐plane strain, κ is curvature, *A* is surface area, $Y=\frac{Eh}{1-\nu^{2}}$ is the stretching rigidity and $B=\frac{Eh^{3}}{12(1-\nu^{2})}$ is the bending rigidity, *E* is Young's modulus, *h* is thickness of plate, *v* is Poisson's ratio. Because bending stiffness scales with the cube of thickness, thin, soft skins preferentially deform out of plane rather than sustain in‐plane stretching. As a result, even small strain mismatches can produce significant thickness changes in compliant systems [195, 197]

In layered and architected soft skins, thickness morphing is also driven by strain mismatch between adjacent layers or spatially patterned actuation fields. For a bilayer structure, the resulting curvature can be approximated as:

(5) $$\kappa \propto \frac{\Delta \varepsilon }{t}$$

where Δε is the mismatch strain and *t* is the total thickness. This scaling highlights a key design principle: reducing thickness or increasing strain mismatch enhances curvature and amplifies out‐of‐plane displacement. More general formulations account for modulus and thickness ratios between layers, which further govern deformation amplitude and profile [198]. Beyond uniform bending, nonuniform or localized actuation produces spatially varying strain fields that give rise to wrinkling, buckling, and complex 3D morphologies. These deformation modes follow the same underlying mechanics, where the final configuration is determined through minimization of elastic energy under geometric compatibility constraints [48, 199]. Consequently, thickness morphing across different actuation strategies can be understood within a unified framework in which in‐plane strain is converted into curvature and surface‐normal displacement. This mechanics‐based perspective provides a foundational link between material response, structural design, and actuator performance.

### 4.6 Physical Constraints and Failure Mechanisms in Thickness‐Morphing Soft Skins

The performance limits of the thickness morphing soft skins are fundamentally governed by underlying physical constraints. A primary constraint arises from the scaling of actuation stress with material properties and driving fields. For electrostatic systems, the generated pressure follows the Maxwell stress relation:

(6) $$\sigma ∼\varepsilon E^{2}$$

where ε is the permittivity and *E* is the electric field. This quadratic dependence implies that large actuation stresses require high electric fields, which are ultimately bounded by dielectric breakdown. As a result, the achievable deformation is fundamentally limited by material dielectric strength, creating a trade‐off between actuation amplitude and reliability.

In thermally‐driven systems, deformation is governed by thermal expansion:

(7) ε∼αΔT

where α is the thermal expansion coefficient. The resulting strain is typically small, requiring large temperature changes to achieve significant deformation. This introduces intrinsic limitations related to energy efficiency, response time, and thermal fatigue, particularly under cyclic operation.

Fluidic and hydrogel‐based systems are constrained by transport phenomena. The actuation speed is governed by diffusion or fluid flow, which scales as:

(8) $$t∼\frac{L^{2}}{D}$$

where *L* is the characteristic length and *D* is diffusivity. This quadratic scaling imposes severe limitations on response time as it increases with device dimensions, which restricts the scalability for large‐area systems.

Beyond actuation limits, mechanical failure mechanisms play a critical role in defining operational boundaries. Repeated thickness modulation induces cyclic strains that can lead to fatigue, crack initiation, and interfacial delamination, particularly in multilayer architectures. These effects are governed by strain amplitude, modulus mismatch, and adhesion energy between layers. In soft composites, large strain gradients can also generate stress concentrations that accelerate material degradation. Another fundamental limitation arises from the competition between bending and stretching energies in thin structures. While thin skins favor bending due to low bending stiffness, excessive deformation can introduce in‐plane stretching, significantly increasing energy cost and potentially leading to mechanical instability such as wrinkling or snap‐through. These instabilities, while sometimes exploited for functionality, can also result in loss of control or structural failure if not properly managed.

## 5 Integrated Architectures and Functional Systems

While individual actuation mechanisms and material platforms provide the building blocks for thickness‐morphing soft skins, practical deployment in haptic interfaces, wearable systems, and unstructured environments requires system‐level integration. Factors such as fabrication scalability, mechanical robustness, sensing integration, power delivery, and closed‐loop control critically influence performance and reliability beyond what can be inferred from isolated actuator demonstrations.

Fabrication complexity and scalability are particularly important for large‐area or high‐density soft skins. Actuators compatible with scalable manufacturing techniques including direct ink writing (DIW), soft lithography, thermoforming, and additive manufacturing are generally favored for practical implementation [135, 200, 201, 202, 203]. In constrained environments, 3D and 4D printing enable the realization of intricately folded, deployable, or architected skins with programmable out‐of‐plane morphing, offering greater functional versatility than bulk‐fabricated components [120, 190]. Mechanical robustness, including fatigue resistance, tear strength, and cyclic durability, is also essential for sustained operation. While SMAs and certain composite actuators exhibit superior longevity compared to hydrogels or electroactive polymers, they may compromise softness or reversibility; bilayer and Janus architectures offer an effective compromise between durability and compliance [119, 190].

Increasingly, integrated sensing and actuation has emerged as a defining requirement for intelligent soft skins. Co‐fabrication of strain, pressure, or temperature sensors enables closed‐loop control, proprioceptive feedback, and adaptive morphing behavior without increasing wiring or footprint. Materials exhibiting piezoresistive, piezoelectric, or triboelectric properties further support multifunctionality within a single deformable layer. In parallel, growing emphasis on sustainability and biocompatibility has motivated the development of Sustainable Soft Actuators (SSAs), including degradable, recyclable, and energy‐efficient systems that align with circular‐economy principles [204, 205, 206].

This section examines integrated and hybrid architectures that combine actuation, sensing, and control to realize functional thickness‐morphing soft skins. Together, these systems illustrate how the technologies discussed in Sections 3 and 4 can be translated into deployable, feedback‐rich interfaces capable of safe, adaptive operation in real‐world environments.

### 5.1 Integrated Sensing and Actuation

For soft skins that reversibly shrink and expand in thickness, integrating sensing and actuation within the same material system is essential for achieving closed‐loop control, perceptually consistent haptic feedback, and adaptive operation in unstructured environments. Unlike conventional rigid systems where sensing and actuation are often spatially separated, soft out‐of‐plane actuators benefit from co‐located or intrinsically coupled sensing, enabling real time measurement of deformation, contact force, or dynamic loading without compromising compliance or form factor.

This type of integration has been demonstrated using soft electromagnetic systems. As an example, a soft electromagnetic actuator with embedded sensing capability, combined intrinsically stretchable conductors, pressure‐sensitive conductive foams, and stretchable magnetic composites [116]. The actuator achieved displacement sensitivity spanning from approximately 0.1 to 1000 nm over a frequency range of 5–3000 Hz, illustrating broadband responsiveness suitable for both static and dynamic out‐of‐plane interactions. Similarly, a soft electromagnetic actuator capable of compressing by more than 50% of its initial dimension has been presented with internal strain‐sensing elements [207]. The actuator operated at frequencies up to 200 Hz during contraction and expansion, enabling feedback‐rich thickness modulation under cyclic loading. Another example is the array‐based haptic systems [208] comprising of an 8 × 12 array of haptic actuators, with a sensor integrated on top of each actuation element. The system employed dielectric elastomer actuation and operated over a frequency range of 0–150 Hz, delivering forces up to 50 mN to the human hand under driving voltages of up to 3.5 kV. The co‐location of sensing and actuation at each taxel enabled localized feedback and spatially resolved control, critical for scalable tactile displays and interactive soft skins.

We have previously demonstrated a flexible actuator–sensor unit termed SensAct (Figure 8a), in which actuation and tactile sensing are seamlessly integrated as a single device [13]. The system consists of a flexible electromagnetic actuator with a soft, squishy touch sensor laminated on top, forming a single monolithic device capable of simultaneous sensing and actuation. The tactile sensor was fabricated using custom graphite paste deposited on a miniature permanent magnet and encapsulated in Sil‐Poxy, providing compliant force and displacement sensing. The actuator itself comprises a flexible coil (∼15 µm thick with 42 turns) fabricated on a polyimide substrate using a lithographie galvanoformung abformung (LIGA) micromolding process. This architecture enables reversible expansion and contraction (squeeze) modes, with operational frequencies ranging from 10 to 200 Hz and out‐of‐plane displacements of up to about 200 µm.

**FIGURE 8:**
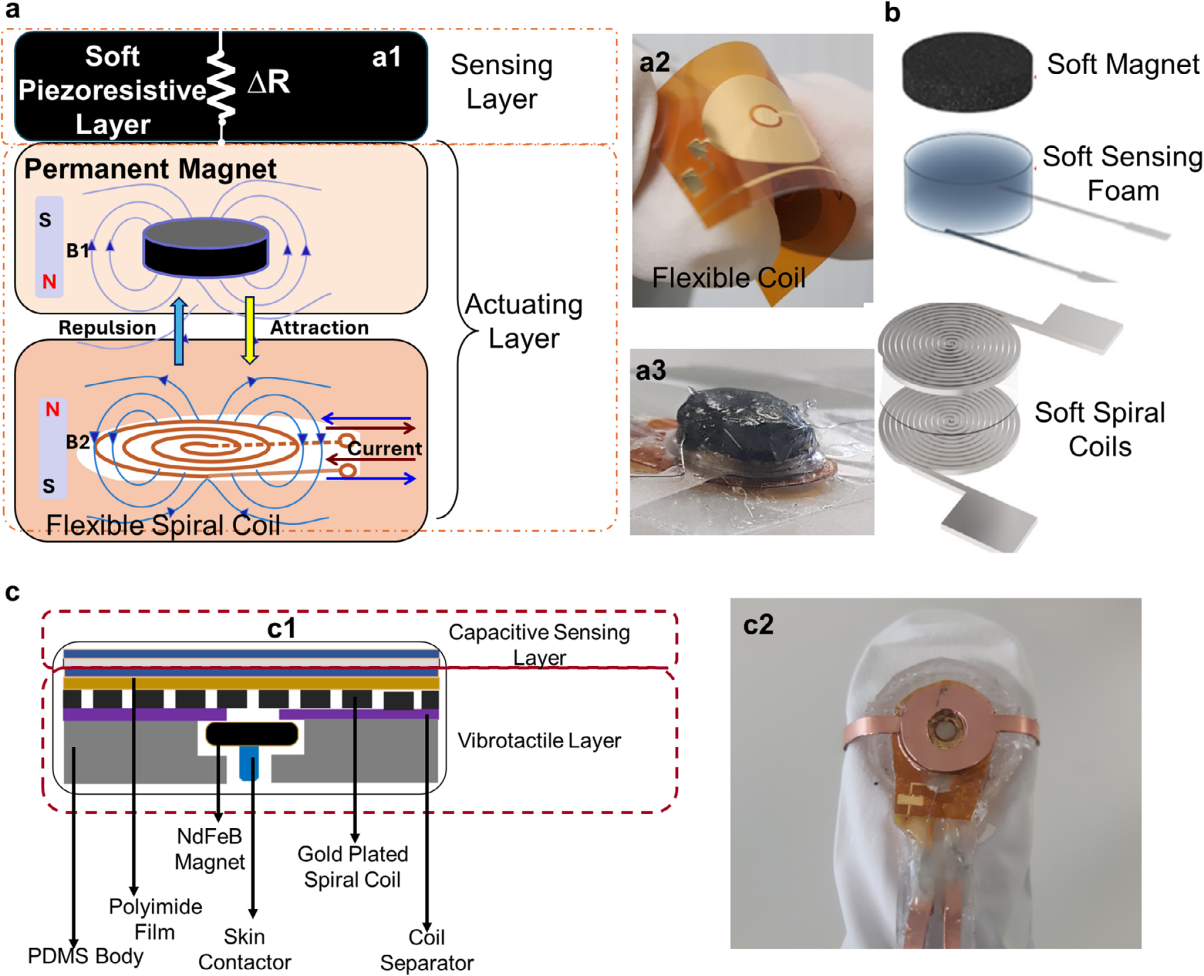
(a) Sensact—a soft device with seamlessly integrated piezoresistive sensor and electromagnetic actuator. Adapted under the terms of the Creative Commons Attribution License [13]. Copyright 2021, The Authors. (a1) the structure and working mechanism of SensAct (a2) the fabricated flexible coil for electromagnetic actuation in Sensact (a3) the fabricated SensAct with integrated sensor and actuator (b) Electromagnetic actuator with integrated soft magnet, spiral coils and sensor. Adapted under the terms of the Creative Commons Attribution 4.0 International License [116]. Copyright 2023, The Authors. (c) Tacsac—an electromagnetic actuator with integrated capacitive sensor for simultaneous tactile sensing and actuation, (c1) the structure of Tacsac (c2) the fabricated Tacsac device with integrated capacitive sensor and electromagnetic actuator for wearable application. Adapted under the terms of the Creative Commons Attribution 4.0 International License [209]. Copyright 2020, The Authors.

An earlier demonstration of this integration of sensor and actuator was demonstrated through Tacsac (Tactile Sensor and actuator, Figure 8c), a dual‐function wearable tactile interface developed to support communication for deafblind users [209]. Tacsac combined a flexible planar capacitive metal–insulator–metal (MIM) touch sensor with a soft electromagnetic vibrotactile actuator, vertically stacked into a single skin‐contacting device. The actuator employed a flexible coil fabricated on a 50 µm polyimide substrate using LIGA micromolding and embedded with a permanent magnet in PDMS, enabling localized vibrotactile stimulation within the human perceptual frequency range of 10–200 Hz. The system achieved a resonance frequency of 60–70 Hz and a maximum out‐of‐plane displacement of approximately 0.377 mm at 180 mA, while the capacitive sensing layer maintained low‐noise touch detection even during active vibration. This architecture enabled reliable bidirectional communication through a single wearable device, representing a significant step toward integrated tactile sensing and feedback systems.

SensAct builds directly on this foundation while advancing integration depth, mechanical compliance, and functional coupling between sensing and actuation. Whereas Tacsac employed a stacked but functionally discrete sensing and actuation architecture, SensAct integrates a squishy, deformable tactile sensor directly into the actuation interface itself, enabling true co‐located sensing and thickness‐modulating actuation at the same tactile point. By embedding the sensing element within the compliant actuation structure rather than placing it as a separate planar layer SensAct improves mechanical conformity, enhances contact intimacy, and enables richer interpretation of local deformation during out‐of‐plane expansion and contraction. In addition, SensAct supports bidirectional squeeze and expansion modes with reduced structural complexity, offering a clearer pathway toward skin‐like, shrinkable and expandable out‐of‐plane actuator arrays with embedded proprioception. In this sense, SensAct represents a functional evolution from stacked multimodality toward material‐level integration, which is essential for scalable, perceptive soft skins capable of closed‐loop interaction in unstructured environments. Further performance enhancement such as increased displacement or force output can be achieved by increasing coil thickness, turn count, or magnetic field strength, without altering the fundamental integration strategy. Likewise, softness of device can be enhanced by using soft magnets instead of rigid permanent magnets. More broadly, these examples highlight how integrating sensing directly into thickness‐morphing actuators enables not only closed‐loop haptic control but also adaptive behaviors such as contact detection, load compensation, and perceptual calibration. As soft skins transition from laboratory demonstrations to field‐deployable systems, integrated sensing and actuation will be indispensable for ensuring localized controllability, robustness, safety, and autonomy.

### 5.2 Hybrid and Multimodal Actuators

Hybrid and multimodal actuators combine multiple actuation stimuli or material platforms within a single system to overcome the limitations of individual approaches and expand the achievable performance envelope of thickness‐morphing soft skins. Rather than optimizing a single metric, such systems exploit complementary mechanisms to balance stroke, force, bandwidth, efficiency, and controllability requirements that are often mutually exclusive in monolithic designs.

A prominent class of hybrid systems couples electrostatic actuation with fluidic amplification, translating high‐voltage electrostatic attraction into hydraulic pressure capable of generating large out‐of‐plane displacement. Examples include hydrostatically coupled DEAs and electrostatic zipping architectures such as HAXELs, which combine compliant elastomers, liquid dielectrics, and flexible electrodes to achieve millimeter‐scale thickness modulation with enhanced force output [127, 130]. These designs decouple electrical input from mechanical output, enabling scalable arrays for haptic displays.

Electromagnetic–mechanical hybrids form another important category. Soft electromagnetic actuators embedded within elastomeric bodies or combined with hydraulic elements leverage magnetic forces for rapid actuation while preserving compliance. Systems integrating electromagnetic attraction with fluid‐mediated force transmission demonstrate both high output force and bistable or latching behavior, making them suitable for thickness‐morphing skins requiring sustained deformation with minimal power draw [137, 138].

Thermal–material hybrids further expand functionality by integrating shape‐programmable materials with localized heating or cooling elements. For example, LCEs combined with resistive or thermoelectric layers enable reversible out‐of‐plane morphing with improved cycle rates and spatial control [162], while SMP–elastomer composites provide deployable architectures with enhanced durability [161]. Electro‐pneumatic and electro‐thermal tactile actuators similarly combine electrical control with fluidic or thermal transduction to improve precision and robustness in haptic interfaces [157].

Although hybrid actuators introduce additional design complexity, they offer a powerful pathway toward application‐specific optimization, particularly in unstructured or constrained environments where adaptability and robustness are paramount. As fabrication and integration strategies mature, hybrid and multimodal systems are expected to play a central role in next‐generation soft skins capable of programmable thickness morphing and intelligent interaction.

### 5.3 Morphological Programming and Fabrication of Thickness‐Morphing Soft Skins

At the system level, morphological programming denotes the deliberate design of geometry, material layout, compliant constraints, and fluid or field routing such that a soft actuator's physical structure directly maps simple inputs to a desired out‐of‐plane deformation. For shrinkable and expandable soft skins, this approach enables individual pixels, tiles, or columns to rise, deform, and relax on demand with predictable height, curvature, bandwidth, and force, without relying on complex control algorithms. Instead, actuation behavior is encoded into the morphology itself, allowing scalable, robust, and energy‐efficient thickness modulation across large areas.

A broad range of thickness‐morphing actuators exemplifies this paradigm. Electrohydraulic zipping cells route dielectric liquid so that an applied electric field pulls a flexible pouch into a dome that fully relaxes when voltage is removed [103, 210]. PVC‐gel electrohydraulic sheets enable dynamic merging and splitting of fluid domains above a printed electrode matrix, allowing local surface height to be repositioned without additional mechanical hardware [85]. Pneumatic laminates define chambers and compliant hinges that inflate into cones, saddles, or columns and collapse flat upon venting [211, 212, 213]. Dielectric elastomer membranes leverage prestretch, thickness control, and electrode geometry to focus electric fields and produce reversible doming deformation [214, 215]. Kirigami and auxetic skins embed cut patterns that pop out under pressure or in‐plane strain and retract with negligible residual height [216]. Printed LCEs program anisotropic contraction so that heating induces cones or saddles that reverse upon cooling [86, 217]. Collectively, these systems demonstrate how morphology can replace control complexity, enabling large‐area tactile arrays and meter‐scale deployable skins that remain thin, quiet, and safe.

Different morphological primitives support distinct functional goals. Doming membranes (shown in Figure 9a) generate smooth normal indentation with millimeter‐scale stroke for tactile pixels and fingertip displays, while multilayer stacks increase displacement without sacrificing thinness [214, 215]. Pouch‐ and bubble‐based inflation (Figure 9b) produces local caps that lift by one to several millimeters per pixel for interactive surfaces and by 15–150 cm per column in architectural tiles, with full collapse upon venting [85, 211, 212]. Electrohydraulic zipping modules (see Figure 9c) enable quiet, high‐density tactile pixels with integrated sensing and fast updates; for example, a 10 × 10 sheet demonstrated closed‐loop control at 200 Hz, actuation up to 50 Hz, and deformation and force sensing resolutions of approximately 0.1 mm and 50 mN, respectively [210]. Hydraulically amplified electrostatic taxels further enable thin, cuttable “sticker‐like” haptic elements delivering 100–800 mN blocked force and 100–850 µm stroke on 10–15 mm pitch, well suited for on‐body haptic interfaces [103]. Bellows and accordion columns provide large stroke at room scale while packing flat for rapid deployment [212]. Kirigami and auxetic pop‐up structures generate directional ridges and cones ideal for programmable textures and mechanically guided height fields [216]. Printed LCE sheets enable reversible thermal pop‐ups for expressive, reconfigurable morphing skins [86, 217]. Serially connected inflatables exploit passive check valves to allow many pixels to share a limited number of supply lines while preserving individual pop‐up and collapse behavior, an important advantage in routing‐constrained systems [213]. Multifunctional PVC‐gel surfaces further demonstrate how quasi‐static lift (∼2.5 mm), strong vibration (up to 300 Hz), and blocked force (∼2 N) can coexist within the same pixel when height and high‐frequency haptics must be combined [85]. In addition, stacked donut‐shaped HASEL doming units have been used to create compliant grippers that pop up under voltage and collapse when deactivated, while planar pouch‐type HASEL actuators drive hinged robotic arms using intrinsic capacitance for self‐sensing of joint state [218].

**FIGURE 9:**
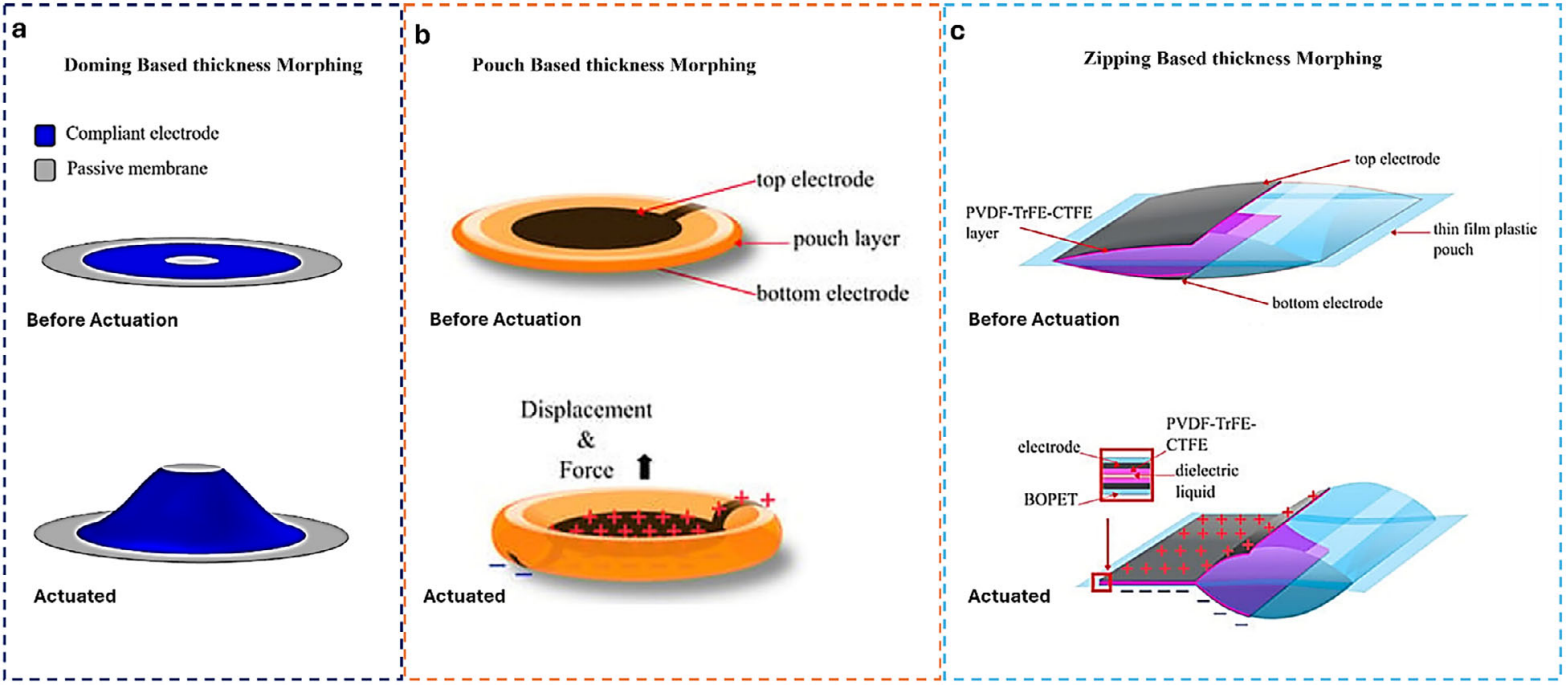
Morphological programming of soft skins. (a) Doming pixel based on a compliant electrode and passive membrane, illustrating electrically induced transition between flat and domed states for localized thickness modulation. Adapted under the terms of the Creative Commons Attribution 4.0 International License from [228]. Copyright 2021, The Authors. (b) Electrohydraulic pouch actuator unit showing voltage‐driven fluid redistribution between electrodes, resulting in controllable out‐of‐plane displacement and force generation. Adapted under the terms of the Creative Commons Attribution 4.0 International License from [229]. Copyright 2025, The Authors. (c) Electrohydraulic zipping cell architecture highlighting multilayer construction and localized zipping‐induced thickness change enabled by dielectric liquid migration. Adapted under the terms of the Creative Commons Attribution 4.0 International License from [229]. Copyright 2025, The Authors.

Fabrication techniques play a central role in enabling these morphologies and directly constrain achievable shape families, bandwidth, and scalability. Soft lithography, molding, and casting yield uniform silicone or acrylic films with thicknesses ranging from tens to hundreds of micrometers, supporting reliable prestretch and lamination for doming dielectric elastomer pixels and multilayer stacks [214, 219]. Heat sealing of polymer films enables rapid formation of pneumatic chambers and electrohydraulic pouches; laser heat sealing in particular accelerates HASEL pouch iteration and facilitates dense hinge patterning for inflatable sheets [211, 218, 220]. Thin‐film processing on PET or polyimide substrates, combined with printed or evaporated compliant electrodes, allows pixels to be integrated onto flexible backplanes and concealed beneath continuous elastomer skins that visually mask pixel boundaries [210, 221]. Additive manufacturing further expands morphological programmability; direct ink writing and inkjet printing, pattern‐compliant electrodes and elastomer layers for monolithic doming elements, while also enabling anisotropic printing of LCEs with spatially programmed director fields for reversible out‐of‐plane morphing [86, 217, 222, 223, 224]. Liquid metal conductors provide highly stretchable, low‐impedance interconnects that tolerate large strains and localized failures in dense arrays, with vacuum‐assisted microchannel filling and embedded‐liquid‐metal elastomers simplifying routing at fine pixel pitch [225, 226, 227]. At large scales, serially connected inflatables reduce valve count and tubing complexity while preserving per‐pixel pop‐up and collapse, and modular tiles demonstrate columns that rise from 15 to 150 cm and stow flat after venting [212, 213]. Together, these fabrication strategies underscore how morphological programming (Figure 9) links material processing directly to out‐of‐plane shape control, performance, and scalability.

Together, the integrated architectures discussed in this section illustrate how actuation stimuli and material platforms can be combined to realize intelligent, feedback‐rich soft skins capable of reversible out‐of‐plane deformation. Because no single approach simultaneously optimizes stroke, force, bandwidth, efficiency, and scalability, practical implementations often rely on careful trade‐offs or hybridization. To facilitate cross‐comparison among the diverse technologies discussed in Sections 3, 4, 5, Table 2 summarizes the key characteristics of representative approaches for reversible out‐of‐plane thickness morphing. The comparison highlights differences in actuation stimulus, material platform, achievable stroke and force, bandwidth, power requirements, scalability, and practical limitations. Rather than identifying a single optimal solution, the table illustrates how different mechanisms occupy complementary regions of the design space, underscoring the importance of application‐specific selection and hybrid integration.

The comparative analysis in Table 2 highlights the breadth of technological solutions available for reversible thickness morphing in soft skins, as well as the absence of a universally optimal approach. Instead, different actuation stimuli and material platforms occupy complementary regions of the design space, each excelling along specific performance dimensions while facing distinct limitations. These trade‐offs underscore several unresolved challenges that must be addressed to transition thickness‐morphing soft skins from laboratory prototypes to robust, field‐deployable systems.

## 6 Performance Metrics, Scalability, and Deployment Readiness of Thickness‐Morphing Soft Skins

Soft actuators that reversibly expand or contract out of plane enable large geometric transformations while preserving intrinsic compliance, contact safety, and adaptability. However, their functional performance varies significantly across material systems, actuation mechanisms, and architectural implementations. Conventional soft actuators, including bending‐based systems such as DEAs and ionic polymer–metal composites, as well as in‐plane stretching systems such as pneumatic networks and hydrogel actuators, have demonstrated substantial capabilities in shape change and force generation. Nevertheless, they do not provide direct control over surface‐normal displacement. In bending‐based systems, out‐of‐plane motion arises indirectly through curvature, leading to geometrically coupled deformation, whereas in‐plane stretching systems are fundamentally restricted to planar expansion.

In contrast, thickness‐morphing systems enable direct and localized control of surface‐normal displacement, representing an independent actuation mode particularly suited for applications requiring programmable surface topography. To highlight these distinctions, Table 3 presents a comparative analysis of representative conventional soft actuators and thickness‐morphing systems across key performance metrics, including stroke, force output, response speed, energy efficiency, and autonomy.

**TABLE 3:** Comparison of conventional soft actuators and thickness‐morphing systems.

Actuation modes	Representative Systems	Stroke (surface‐normal)	Force output	Response speed	Energy efficiency	Autonomy	Key limitations	Rep. Refs.
Out‐of‐plane bending	Dielectric elastomer bending actuators, ionic polymer–metal composites (IPMCs), and thermal bimorphs.	Indirect (curvature‐induced and typically sub‐mm to mm‐scale equivalent displacement)	mN–N	Fast (ms–s for DEAs), slower for thermal systems	Moderate–high (DEAs) and low (thermal)	Electrically‐driven but often constrained by structure	Vertical displacement coupled to curvature and no independent thickness control	[238, 239]
In‐plane stretching	Pneumatic networks (PneuNets), hydrogel actuators, and electrostatic stretch systems.	None (planar deformation only)	Low–10^2^ N (pneumatics)	Slow–moderate (<10 Hz typical)	Moderate (pneumatic losses) and low (hydrogels)	Often tethered (fluidic supply or environmental dependence)	Cannot generate localized surface‐normal displacement	[84, 92, 240]
Thickness morphing	Dielectric elastomer domes, electrohydraulic (HASEL) actuators, fluidic domes, and electromagnetic soft actuators.	Direct and localized (∼0.1 mm to cm‐scale depending on mechanism)	mN–10^2^ N (mechanism‐dependent)	Broad range (Hz to kHz depending on modality)	Moderate–high (electrical systems) and moderate (fluidic)	High potential for untethered and integrated systems	Trade‐offs between speed, force, and efficiency; emerging design space	[218, 241, 242]

In the remainder of this section, the technologies reviewed in this paper are evaluated using commonly reported performance metrics and, where appropriate, compared with rigid actuation benchmarks (Table 4). The discussion focuses exclusively on systems that explicitly realize thickness modulation rather than purely in‐plane deformation, including doming dielectric elastomer membranes, electrohydraulic domes and taxels, fluid‐driven pouch and bellows architectures, photothermal and photoisomerizable sheets that undergo out‐of‐plane buckling, hydrogel‐ and osmotic‐driven swelling compartments, and biohybrid plates that deform through coordinated tissue contraction.

**TABLE 4:** Performance summary of soft skins with reversible thickness morphing.

Technology class	Primary performance strength	Best‐in‐class metrics	Typical operating regime	Deployment readiness	Most suitable applications	Key constraints	Rep. Refs.
Dielectric elastomer domes	High bandwidth with large and normalized stroke	kHz‐scale thin‐film response; >10^2^ J kg^−^^1^ specific energy	Fast, electrically‐driven, and moderate force	High (arrays demonstrated)	Tactile displays, shape‐changing surfaces, and adaptive optics	High voltage (kV) and dielectric breakdown	[230, 243, 244, 245, 246, 248]
Electrohydraulic (HASEL and zipping) actuators	Balanced stroke, force, and speed	Muscle‐like stress; mm‐scale doming; tactile bandwidth	Fast electrical actuation with fluid mediation	High (large‐area arrays demonstrated)	Haptic skins, object manipulation, and wearable interfaces	High voltage (in kV) and sealing complexity	[218, 231]
Pneumatic/Hydraulic skins	Very large force and stroke	cm‐scale deformation; >10^2^ N force	Pressure‐driven and moderate speed	Moderate (tethered systems common)	Soft grippers, morphing cushions, and large‐area skins	Pumps, routing and slower dynamics	[232, 237]
Hydrogel‐based hydraulic domes	Fast volumetric expansion at low voltage	>1 N force; <1 s response	Fluid‐driven swelling or pressurization	Moderate	Biomedical skins and soft interfaces	Dehydration and material aging	[237, 247, 254]
Osmotic forward‐osmosis domes	Ultra‐low‐power force generation	>20 N at mW‐level power	Quasi‐static and diffusion‐limited	Low–moderate	Long‐term shape holding and silent actuation	Slow response and membrane fouling	[188, 255]
Thermal SMA plates/laminates	Extremely high blocking force	Highest force density among soft systems	Thermally‐driven and slow cycling	Moderate	Load‐bearing morphing panels	Fatigue and low efficiency	[234, 235, 251]
LCE sheets (thermal/optical)	Programmable 3D shape morphing	Large strain; complex curvature	Thermally or optically‐driven	Moderate–high (printing enabled)	Shape displays and reconfigurable skins	Thermal gradients and slow relaxation	[243]
Biohybrid plates and fins	Smooth biomimetic curvature	Continuous arching motion	Chemically powered and low frequency	Low	Bio‐interfaces and research demonstrators	Lifetime and environmental control	[192, 193]

### 6.1 Out‐of‐Plane Stroke and Geometric Amplification

A practical way to compare out‐of‐plane stroke is the dome height‐to‐span ratio, often written as h/D for circular domes, or as the maximum normal displacement normalized by thickness in multilayers. Dielectric elastomer devices can morph into domes or saddles by programming electrode mesoarchitecture, with measured dome heights that scale with applied field and layout while preserving thin sheet form factors [230, 243, 244]. Recent dielectric elastomer formulations have reached very large area strains at reduced fields, which translates to high out‐of‐plane deflections for a given footprint [245].

Electrohydraulic actuators that confine a liquid dielectric beneath a flexible membrane produce large, repeatable out‐of‐plane strokes; early HASEL reports show muscle‐like strain with thin pouch geometries that are easily arranged as arrays of pop‐up elements [218, 231]. Hydrogel and osmotic actuators naturally provide substantial volumetric change; forward osmosis units at centimeter scale achieve dome‐like expansion in minutes with high pressure generation, while hydraulic hydrogels achieve very fast bulging when driven by on‐board fluid [188, 237]. Biohybrid tissues on thin scaffolds can produce millimeter‐scale arching of plates and fin‐like sheets, but their shrinkability is limited by tissue prestrain and scaffold mechanics [192, 193].

Compared to rigid stacks such as piezoelectric columns or voice‐coil stages, which offer micrometer‐ to sub‐millimeter strokes in compact footprints, soft domes provide much larger normalized out‐of‐plane strokes but at lower absolute stiffness [246].

### 6.2 Force Output and Load‐Bearing Capability

Force generation in thickness‐morphing actuators follows the working stress or pressure of the active medium. Electrohydraulic HASEL devices report actuation stresses on the order of ∼10^−^^1^ MPa while sustaining large strains, enabling thin domes and shells that lift substantial payloads relative to their mass [218, 231]. Dielectric elastomers combine large strain and high specific energy; blocking force increases rapidly with thickness and prestretch, and improved dielectrics have pushed specific energy above 200 J kg^−^^1^ under moderate fields [230, 245].

Osmotic actuators can generate high forces from small volumes because osmosis creates high effective pressure; a 10 mm‐scale forward osmosis actuator produced >20 N at milliwatt electrical input, with actuation over minute timescales [188]. Hydraulic hydrogel actuators exceed 1 N with response times below 1 s when driven by internal fluid pressure [237]. Thermal shape memory alloys configured in plates or laminates offer very high blocking force for size and can lift heavier loads than most soft elastomeric systems, but their duty cycle is constrained by heating and cooling dynamics [235]. Biohybrid plates and fins move modest payloads for their mass and remain below electrohydraulic and pneumatic systems in peak force output [192, 193].

Rigid benchmarks such as piezo stacks deliver very high force and stiffness at small stroke, and voice‐coil stages deliver moderate force at larger stroke; both exceed most soft domes in stiffness‐limited payloads but cannot match the safe, large deformation of soft out‐of‐plane devices [246].

### 6.3 Bandwidth and Dynamic Response

Electric‐field‐driven elastomers are among the fastest soft actuators, as their operation is governed by electrostatic rather than diffusive or thermal processes. Thin dielectric elastomer films exhibit frequency responses extending into the kilohertz range, while out‐of‐plane domes and shells typically operate up to ∼10^2^ Hz in compact formats [230, 243, 246]. Electrohydraulic domes inherit similar electrical dynamics, with added fluid inertia, and can achieve tactile‐relevant bandwidths while maintaining large stroke [218, 231].

Thermally‐driven SMAs are generally limited to ∼1 Hz due to heating and cooling transients [235]. Forward‐osmosis actuators operate over minutes by design [188], whereas hydraulic hydrogel systems overcome diffusion limits and achieve sub‐second response for dome‐like bulging [237]. Electrified hydrogels driven by ionic currents can reach response times below 1 s at voltages under 3 V [247]. Biohybrid tissues operate on the order of 1 Hz due to intrinsic myocyte physiology and culture constraints [192, 193]. In comparison, rigid piezo stacks provide the highest practical bandwidth at small stroke, and voice‐coil actuators offer high bandwidth at larger strokes but with reduced stiffness [246].

### 6.4 Energy Efficiency and Specific Power

Electrohydraulic and DEAs achieve high specific power because electrical input is converted directly into mechanical work with minimal thermal lag. Dielectric elastomer systems report specific energies exceeding 10^2^ J kg^−^^1^ and high specific power at modest frequencies [230, 245]. Electrohydraulic devices similarly achieve muscle‐like specific power with low current at high voltage [218, 231].

Osmotic actuators stand out for ultra‐low electrical power consumption: forward‐osmosis domes deliver forces above 20 N while consuming only milliwatt‐level electrical power to maintain concentration gradients [188]. SMAs provide high instantaneous power density during heating but suffer from poor cycle efficiency due to Joule heating and cooling losses [235]. Electrified hydrogels operate at low voltages but may be limited by ionic transport losses and electrolyte management [247]. Biohybrid actuators derive energy chemically, yet their overall energy budget is dominated by life‐support requirements rather than mechanical output [193]. Relative to rigid systems, piezo stacks are highly efficient within a narrow stroke envelope, while voice‐coil motors are efficient when paired with optimized drivers; neither matches osmotic systems for ultra‐low‐power quasi‐static holding [188, 246].

### 6.5 Durability, Fatigue, and Reliability

DEAs can exceed 10^6^ cycles when operated below breakdown thresholds with appropriate materials and prestretch, with recent work emphasizing durability gains through dielectric and electrode design [230, 248]. Key failure modes in soft thickness‐morphing actuators include dielectric breakdown, electromechanical instability, and electrode delamination under repeated large‐strain cycling. To address these limitations, a range of mitigation strategies has been developed, including dielectric layer optimization, defect‐minimizing fabrication approaches, compliant electrode encapsulation, and the incorporation of self‐healing polymer networks. These approaches reduce localized electric field concentrations and help maintain interfacial integrity over extended operational lifetimes [249]. Electrohydraulic HASEL actuators exhibit an intrinsic advantage in this context, as dielectric breakdown events can be partially mitigated through self‐healing behavior enabled by liquid dielectric redistribution, allowing continued operation after failure and extending device lifetime [218, 231]. Nevertheless, long‐term durability remains constrained by polymer shell fatigue, seal leakage, and stress concentration at electrode boundaries. Recent advances have therefore focused on improved shell elastomers, reinforced sealing strategies, and optimized fluid–electrode configurations to reduce fatigue‐induced rupture and charge loss [250].

SMAs are similarly limited by both functional and structural fatigue, with operational lifetimes strongly dependent on stress amplitude, thermal cycling range, and actuation protocols. Without careful design, degradation can occur within 10^3^–10^4^ cycles [234, 251]. Mitigation strategies include restricting operation to limited transformation strain windows, implementing effective thermal management to control phase transitions, and employing closed‐loop control schemes to prevent overheating and cyclic overstress [252, 253]. For hydrogel‐based systems, durability is governed by material formulation, interfacial adhesion, and hydration stability. While tough hydrogel networks can sustain thousands of large‐deformation cycles, osmotic systems may suffer from fouling, solvent loss, or reduced transport efficiency over time [254, 255]. Dominant failure mechanisms include dehydration, network fatigue, and interfacial debonding. These challenges are typically addressed through crosslink density optimization, moisture‐retaining encapsulation layers, and the integration of self‐healing polymer chemistries [256, 257]. Biohybrid tissues can operate stably for weeks to months under pacing but eventually remodel and decline in output [193]. Rigid benchmarks such as piezo stacks routinely exceed 10^6^ cycles under appropriate drive conditions [246]. Overall, some of the mitigation strategies discussed in this section, such as reinforced polymer interfaces, compliant encapsulation, and optimized bonding, represent targeted interfacial engineering approaches which are necessary to enhance structural stability and cyclic durability across all layered and multifunctional composite soft skins.

### 6.6 Scalability, Crosstalk, and Deployment Readiness

Scalability and deployment readiness are critical for translating thickness‐morphing soft skins from laboratory demonstrators to large‐area tactile displays, wearable interfaces, and field‐deployed robotic systems.

Programmable electrode patterns in dielectric elastomers enable precise control of Gaussian curvature, suppressing unwanted buckling and reducing crosstalk across actuator arrays by localizing strain [243, 244]. Electrohydraulic domes and taxels benefit from hydraulic isolation between cells, reducing mechanical coupling compared with pneumatic skins; electrical failure in one cell typically causes fluid redistribution that limits damage propagation [218, 231].

Thermal shape memory laminates and LCE sheets achieve complex, repeatable morphologies through director or laminate programming, although thermal gradients and hysteresis can cause overshoot without careful control [235, 243]. Hydrogel and osmotic domes may suffer from diffusion‐driven nonuniformity or membrane fouling unless channels and interfaces are carefully engineered [188, 237]. Biohybrid sheets can drift in shape as tissues remodel but excel at generating smooth, continuous curvature when patterned appropriately [192, 193].

Rigid stacks offer the highest per‐element shape fidelity but require discrete joints or linkages to approximate continuous morphing surfaces, limiting their scalability for large‐area adaptive skins [246].

Table 4 synthesizes where each out‐of‐plane technology generally excels, using representative values from peer‐reviewed sources. Soft thickness‐modulating actuators outperform rigid systems in geometric adaptability, contact safety, large normalized stroke, and the ability to realize continuous surface morphing such as domes, saddles, and traveling waves [243, 244]. Electrohydraulic and dielectric elastomer devices increasingly close the gap with rigid actuators in specific power and bandwidth while retaining softness [218, 230, 245]. Osmotic and hydrogel systems dominate applications requiring silent, ultra‐low‐power quasi‐static shape holding [188, 237].

Rigid actuators remain superior in absolute stiffness, nanometer‐scale precision, and extreme bandwidth. Shape memory plates offer high blocking force but lag in efficiency and duty cycle [235]. Biohybrid tissues provide unique biomimetic motion but remain slower and weaker than electrostatic or hydraulic systems and require substantial biological support [192, 193]. Collectively, these comparisons define the practical performance envelope of thickness‐morphing soft skins and motivate the challenges and future directions discussed in the following section.

## 7 Applications of Thickness‐Morphing Soft Skins

Soft skins that reversibly expand and shrink out‐of‐plane enable a class of interactions that cannot be readily achieved with rigid or purely in‐plane deformable systems. By locally modulating thickness, curvature, and contact force, these actuators transform compliant surfaces into functional interfaces capable of conveying information, adapting shape, and interacting safely with complex environments. Crucially, the same fundamental capability, controlled, reversible out‐of‐plane deformation supports applications spanning multiple length scales, from millimeter‐scale haptic pixels to meter‐scale deployable structures and autonomous robots operating in cluttered or occluded terrain.

This section surveys representative application domains in which thickness‐modulating soft skins provide decisive advantages. Rather than exhaustively cataloging devices, the discussion emphasizes how specific actuation mechanisms and morphological design choices map onto application requirements such as stroke amplitude, bandwidth, force output, scalability, and robustness. The applications are grouped into haptics, programmable shape‐changing interfaces, large‐area deployable skins, navigation in constrained environments, and emerging rehabilitation and assistive technologies.

### 7.1 Haptic Interaction

Haptic feedback benefits directly from actuators that raise and lower a compliant surface with controllable stroke and bandwidth while remaining thin, quiet, and safe to touch. Shrinkable and expandable out‐of‐plane soft actuators meet this need because each pixel returns to a low‐profile state when power is removed yet can deliver normal indentation and vibration on demand. The principal device families include electrohydraulic zipping cells that dome out‐of‐plane, pneumatic bubbles and skins that inflate and deflate, and dielectric elastomer membranes engineered to produce localized doming. These approaches have been demonstrated on fingertips, across the hand and forearm, on the torso, and in table‐ or room‐scale panels [103, 210, 214, 215, 258, 259, 260].

The examples include a 10 × 10 electrohydraulic shape display, which integrates actuation, sensing, and control within each cell and achieves closed‐loop update rates of 200 Hz, mechanical actuation up to 50 Hz, deformation sensing at 0.1 mm resolution, and force sensing at 50 mN resolution. The system produces smooth, quiet normal indentation beneath a continuous elastomer skin, enabling dynamic finger exploration and tactile interaction [210]. A complementary electrohydraulic surface based on a polyvinyl chloride (PVC) gel composite achieves a thin profile of approximately 1.5 mm while combining millimeter‐scale height change with high‐frequency vibration on the same pixel. Reported blocked forces approach 2 N with vibrational forces up to 2.4 N over 0.1–300 Hz, and a 5 × 5 matrix renders letters, textures, and object transport on the morphed surface [85].

Soft pneumatic arrays (SPA) provide thin, body‐conforming haptic feedback over large areas. The SPA skin uses silicone bubbles of 3–4 mm diameter that inflate 0.8–1 mm out‐of‐plane and deliver blocked forces around 0.3 N with closed‐loop vibrotactile control from 10 to 90 Hz, enabling distributed cues on curved body regions [258]. Hydraulically amplified electrostatic taxels form cuttable haptic “stickers” that adhere to skin or garments. Arrays with pixel sizes from 2 to 15 mm generate blocked forces of 100–800 mN with strokes of 100–850 µm and operate from DC to 200 Hz. Validation across multiple body locations confirms that thin out‐of‐plane taxels scale effectively for wearable haptics [103].

Multilayer DEAs have long been used for tactile displays that indent the fingertip with pixelated normal force at tens to hundreds of hertz while remaining millimeter‐scale in thickness [214, 215]. For compact text interfaces, tubular dielectric elastomer pixels developed for refreshable Braille displays exploit axial contraction to generate dome‐like protrusions under compliant caps and fully relax when the electric field is removed [259, 260].

### 7.2 Programmable Shape‐Changing Interfaces and Displays

Shrinkable and expandable out‐of‐plane actuators are a natural fit for adaptive surfaces that must raise, lower, and locally reshape a compliant skin while remaining thin and safe to touch. Electrohydraulic zipping arrays, pneumatic pouch and bladder systems, and dielectric elastomer membranes dominate this space, offering direct pixel‐level height control with compliant contact and high areal power density [85, 103, 210, 211, 214, 259, 260]. Electrohydraulic zipping arrays provide one of the clearest realizations of a fully soft shape display. This is seen in the PVC gel–based electrohydraulic surface shown in Figure 10a,c,d,e which features seamless redistribution of dielectric fluid above a printed electrode matrix, achieving approximately 2.5 mm of out‐of‐plane morphing at kilovolt drive with strong vibratory output [85].

**FIGURE 10:**
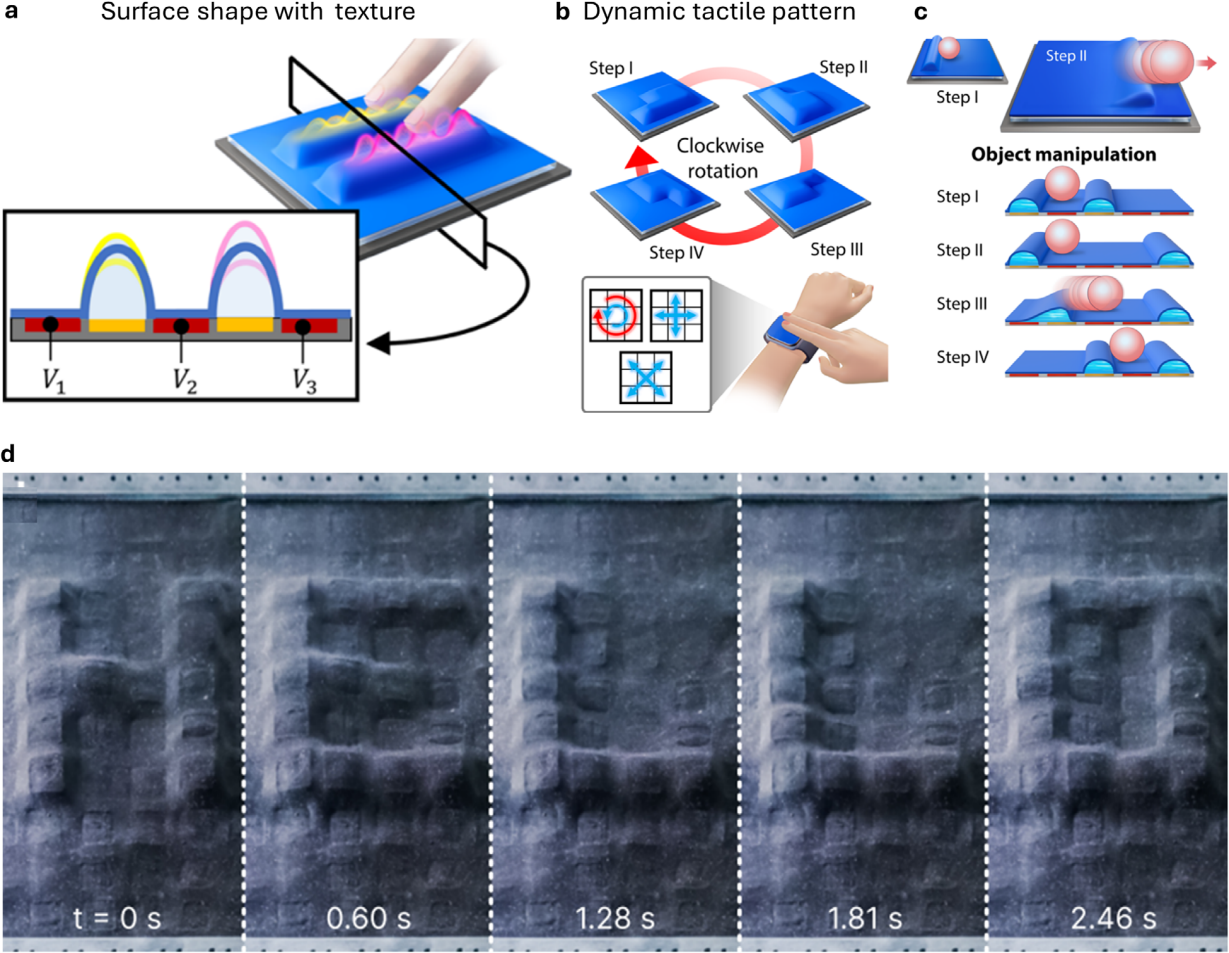
Programmable shape‐changing interfaces and large‐area applications enabled by thickness morphing soft skins. (a) Combined shape and texture rendering through dynamic thickness modulation, enabling surface shape morphing with superimposed tactile cues. Adapted with permission under the terms of the Creative Commons Attribution‐NonCommercial 4.0 International License [85]. Copyright 2024, The Authors; exclusive licensee American Association for the Advancement of Science. (b) Dynamic tactile patterns generated through spatiotemporal actuation sequences, producing directional and rotational haptic feedback. Adapted with permission under the terms of the Creative Commons Attribution‐NonCommercial 4.0 International License [85]. Copyright 2024, The Authors; exclusive licensee American Association for the Advancement of Science. (c) Time‐lapse surface deformation demonstrating rapid and reversible shape morphing across a large active area. Adapted with permission under the terms of the Creative Commons Attribution‐NonCommercial 4.0 International License [85]. Copyright 2024, The Authors; exclusive licensee American Association for the Advancement of Science. (d) High‐speed object conveyance and manipulation enabled by programmed surface morphing and liquid‐mediated inertia effects. Adapted under the terms of the Creative Commons Attribution 4.0 International License [210]. Copyright 2023, The Author(s).

Electrostatic zipping taxels with hydraulic amplification have been specialized into thin haptic stickers suitable for wearable and body‐scale displays. Their shrink–expand behavior allows arrays to be tiled beneath a continuous elastomer skin to create reconfigurable height fields without rigid pin mechanisms [103]. Pneumatic interfaces remain the most scalable route to large‐area shape change. Thin inflatable sheets and heat‐sealed laminates can pop into cones, saddles, and folds upon pressurization and return to flat on venting, enabling interactive wearables, furniture‐scale panels, and room‐scale displays [211, 212, 260]. Serial connection strategies reduce tubing and valve count while preserving per‐pixel expansion and collapse [188].

### 7.3 Large‐Area Deployable Skins and Tactile Arrays

Large‐area applications (Figure 10) benefit from the inherent stowability of shrinkable and expandable out‐of‐plane actuators, which can collapse into flat sheets for transport and deployment. Heat‐sealed inflatable laminates enable meter‐scale panels that repeatedly morph and flatten [211]. Modular inflatable columns such as LiftTiles raise elements from 15 to 150 cm while supporting loads of approximately 10 kg per column, demonstrating architectural‐scale shape modulation that packs flat after use [212].

To reduce control complexity, serially addressable pneumatic arrays connect multiple inflatables through bidirectional check valves, allowing large arrays to be driven with minimal tubing while preserving individual expansion and collapse [213]. To make big arrays tractable without a valve per pixel, *PneuSeries* connects inflatable modules with bidirectional check valves, so inflation and deflation propagate in series. This pattern preserves per unit out‐of‐plane expansion while drastically cutting tubes and drivers for large surfaces. For tactile feedback over large, curved surfaces, pneumatic bubble skins and electrohydraulic taxel arrays provide distributed, compliant out‐of‐plane actuation suitable for robotic skins and wearable interfaces [103, 258].

### 7.4 Navigation in Cluttered, Occluded, and Collapsible Terrains

In cluttered or collapsible environments, the actuator is rarely a standalone component; instead, it is structurally integrated into the robot body. Shrinkable and expandable out‐of‐plane actuation enables locomotion, anchoring, and reconfiguration through volumetric change rather than rigid joints. Here examples include the tip‐everting “vine” robots expand outward at the tip, enabling navigation through rubble and confined voids with minimal friction and safe contact [261, 262, 263]. Radial and axial expansion modules enable peristaltic motion and anchoring in pipes and ducts [264]. Origami and kirigami crawlers flatten to pass narrow gaps and then pop out‐of‐plane to traverse obstacles [265]. Recent modular soft origami platforms with electrothermal actuation compose plug‐and‐play DoFs to squeeze through tight passages and reassemble into body shapes better suited for obstacle negotiation [266]. Inflatable kirigami crawlers exploit cut‐pattern‐induced out‐of‐plane buckling to double contraction versus simple pouches and generate anisotropic friction for directed motion across coarse substrates [267].

Across these systems, controllable out‐of‐plane expansion enables robots to exploit contact rather than avoid it, making them well suited to search‐and‐rescue, inspection, and subterranean exploration [268, 269, 270]. While the literature reports such works as robot systems, the enabling mechanism is often an actuator that changes shape out‐of‐plane with large stroke and controllable stiffness. Practical challenges such as retraction without buckling have led to solutions that restore controllable inversion for backing out of tight voids and revisiting branches [271]. Autonomous behaviors inspired by climbing plants (circumnutation and thigmotaxis‐like contact use) now allow self‐navigating growth in unstructured clutter [272, 273, 274].

In sandy or debris‐filled volumes liable to collapse, volume‐change and eversion enable low‐drag burrowing and easy back driving. Earlier work showed that combining tip‐extension with granular fluidization reduces penetration forces by roughly an order of magnitude versus rigid intruders, critical when tunnels are occluded by caving material [275]. Untethered everting designs extend this concept by continuously everting skins to minimize side friction even when the medium compacts [276]. Recent advances in polymeric ionogels highlight the potential of ultrathin, suspended 3D morphing architectures for rapid and highly sensitive pressure sensing, particularly in aquatic environments. For example, a fluorinated ionogel incorporating tert‐butyl groups and a hydrophobic ionic liquid achieves high optical transparency (96.38%) and enhanced ionic conductivity (1.74 mS cm^−^^1^), enabling pressure detection down to ∼2.9 Pa. When integrated into a dolphin‐inspired untethered robot, this system enables flow sensing and autonomous obstacle avoidance, demonstrating that suspended 3D morphing skins can simultaneously achieve actuation and self‐sensing in complex fluidic environments [277].

Compared to conventional fluid‐driven skins, such architectures offer reduced thickness, simplified sensing integration, and faster out‐of‐plane response. Similarly, suspended e‐skins capable of synergistic touch and pain perception have been developed based on 3D deformation and contact mechanics. These systems can detect extremely small stimuli (∼0.02 Pa), maintain stable performance over thousands of cycles (∼5200), and have been demonstrated on artificial fingers for feedback‐controlled human–robot interaction [278]. Collectively, these advances underscore the promise of suspended 3D morphing architectures as a pathway toward highly sensitive, mechanically compliant, and multifunctional soft skins that integrate actuation and sensing within a unified platform.

### 7.5 Rehabilitation and Communication Interfaces

Thickness‐morphing soft skins are particularly well suited to rehabilitation and assistive communication technologies, where compliance, safety, and adaptability to the human body are paramount. Unlike discrete vibrotactile motors, out‐of‐plane soft actuators provide localized, programmable indentation and pressure while conforming to anatomical curvature and minimizing discomfort during prolonged use. Wearable tactile interfaces can support sensory retraining following stroke, spinal cord injury, or peripheral neuropathy by delivering graded normal indentation and vibration to stimulate mechanoreceptors during motor rehabilitation. Distributed arrays of shrinkable and expandable taxels integrated into gloves, sleeves, or braces can provide task‐specific feedback while preserving freedom of movement. An example, based on Tacsac device described in Section 5.1, exemplifies how integrated sensing and out‐of‐plane actuation can enable bidirectional tactile communication for deafblind users. These devices were integrated in a smart glove to enable bluetooth‐enabled tactile communication for deafblind people using Braille codes [96, 209]. The evaluation with 20 end‐users (10 deafblind and 10 sighted and hearing person) of the tactile interface under standardized conditions demonstrated that users could feel and distinguish the vibration at frequencies ranging from 10 to 200 Hz which is within the perceivable frequency range for the mechanoreceptors in the skin. The results showed that non‐experts in Braille could send and receive within 25 and 55 s, respectively words like “best” and “journal”, with an accuracy of ∼75% and 68%, respectively.

Beyond sensory substitution, thickness‐modulating soft skins can support posture guidance and physical assistance. Localized inflation or doming can apply gentle corrective cues to guide joint alignment, balance, or gait without rigid constraints. Because actuation is distributed and compliant, such systems accommodate natural movement variability and offer advantages over conventional rigid orthotic devices. As integration with physiological sensing and adaptive control improves, soft skins based on out‐of‐plane actuation are poised to play a growing role in personalized rehabilitation and assistive care.

The application examples presented in this section highlight both the versatility and the current limitations of thickness‐morphing soft skins. While compelling demonstrations now exist across haptics, adaptive interfaces, deployable structures, locomotion, and assistive technologies, these systems also expose persistent challenges related to durability, scalability, power delivery, control complexity, and long‐term reliability in real‐world settings. The diversity of application requirements further underscores that no single actuation strategy universally satisfies all use cases. In the following section, we synthesize these challenges and outline key opportunities that must be addressed to transition thickness‐modulating soft skins from laboratory prototypes to robust, widely deployable technologies.

## 8 Outlook: Toward Autonomous, Perceptive, and Deployable Thickness‐Morphing Soft Skins

Soft skins with reversible thickness morphing have progressed rapidly from isolated actuator demonstrations to integrated systems capable of complex, programmable out‐of‐plane deformation. As synthesized throughout this review, thickness modulation enables a unique combination of volumetric reconfiguration, intrinsic compliance, and surface‐level functionality that cannot be readily achieved using rigid mechanisms or purely in‐plane soft actuators. At the same time, the diversity of actuation stimuli, material platforms, and architectural strategies reveals that thickness morphing is not a single technology, but rather an emergent materials design paradigm defined by surface‐normal deformation as a primary functional axis. Translating this paradigm into autonomous, reliable, and field‐deployable systems requires addressing a set of tightly coupled challenges that span materials science, mechanics, energy transduction, and system integration.

### 8.1 Materials Durability Under Cyclic Volumetric Strain

A defining characteristic of thickness‐morphing soft skins is repeated volumetric deformation concentrated along the surface‐normal direction. This deformation mode places unique demands on materials that are not fully captured by traditional metrics used for in‐plane stretchable systems. Cyclic thickness change induces multiaxial stress states, interfacial shear in multilayers, and localized strain amplification at geometric features such as domes, folds, and hinges. As a result, fatigue, delamination, dielectric breakdown, solvent loss, leakage and microcracking often emerge as dominant failure modes, even when in‐plane strains remain moderate. In real‐world settings such as rubble, industrial pipelines, or biological environments, exposure to sharp edges, particulates, biofluids, and chemical contaminants further exacerbates degradation. Soft materials also exhibit nonlinear, hysteretic, and history‐dependent behavior, complicating traditional model‐based estimation and control. Large deformation, viscoelasticity, and fluid–structure interaction further challenge predictive modeling, particularly when buckling, doming, or shell inflation is the intended mode of operation. Machine‐learning and hybrid physics–data‐driven modeling approaches are emerging as credible pathways to handle nonlinear, unpredictable behavior when first‐principles models break down.

Future progress will depend on materials architectures explicitly designed for volumetric cycling, rather than adapted from planar soft actuators. Promising directions include toughened elastomers with engineered energy dissipation, damage‐tolerant architectures, self‐healing dielectrics and gels, interpenetrating polymer networks, and graded or anisotropic composites that redistribute stress through thickness. Intrinsic self‐healing elastomers, ionic networks, and gels capable of autonomously recovering from cuts or pinholes have advanced rapidly [279, 280]. Complementary encapsulation strategies, solvent‐retention techniques, and multilayer barrier designs mitigate drying, swelling, and radiation‐induced degradation are need to address issues that particularly relevant in aerospace, industrial inspection, and biomedical contexts [281]. Importantly, durability must be considered at the system level: interfaces between active and passive layers, electrodes and encapsulants, and fluidic or ionic domains frequently dictate lifetime more strongly than the bulk material properties alone [280].

### 8.2 Energy Efficiency, Power Autonomy, and Untethered Operation

Many high‐performance thickness‐morphing systems particularly electrostatic, electrohydraulic, and fluid‐driven architectures currently rely on external power supplies, kilovolt‐level driving voltages, or off‐board pumps. While acceptable for laboratory demonstrations, these requirements limit autonomy, safety, and scalability, especially in wearable, biomedical, and mobile robotic applications. Achieving untethered operation remains one of the most significant barriers to deployment.

Addressing this challenge requires a shift from actuator‐centric optimization toward energy‐aware material and system design. Strategies include lowering actuation voltages through high‐permittivity and ultra‐thin dielectrics, improving electromechanical coupling efficiency, exploiting mechanical latching or bistability to reduce holding power, and integrating on‐board fluidic or electrochemical energy storage [229, 282, 283]. Biohybrid and osmotic systems highlight alternative pathways in which chemical gradients or metabolic energy directly drive thickness change, albeit with their own integration challenges. Ultimately, autonomous thickness‐morphing skins will require co‐design of materials, architectures, and power electronics to balance stroke, force, bandwidth, and efficiency within realistic energy budgets.

### 8.3 Integration of Sensing, Computation, and Control Through Thickness

A central opportunity enabled by thickness morphing is the ability to embed multiple functions; actuation, sensing, signal transmission, and even computation within the thickness of a soft skin. However, most current systems treat sensing and control as add‐ons rather than intrinsic material properties. Wiring complexity, signal interference, and mechanical mismatch between sensors and actuators often constrain scalability and robustness. One promising direction is the intrinsic integration of sensing within the actuator body. Seamlessly embedded strain, pressure, magnetic, or capacitive sensors preserve compliance while enabling proprioception and contact awareness [284]. The integration of soft actuation with sensory feedback is a critical goal in emerging skin‐like interfaces for robotics, prosthetics, and wearable technologies [64, 285, 286]. By embedding sensing, local computation, and adaptive control directly within the actuator, thickness‐morphing soft skins can begin to exhibit self‐sensing and self‐correcting behavior an essential step toward autonomous operation in unstructured environments.

Future thickness‐morphing soft skins are likely to adopt functionally integrated material systems, in which deformation, sensing, and feedback are co‐located. Piezoresistive, piezoelectric, capacitive, and triboelectric responses can be encoded directly into active layers, enabling proprioception and contact sensing without additional components. Advances in soft electronics, stretchable interconnects, and embedded fluidic or ionic logic further point toward skins that compute and respond locally, reducing reliance on centralized control. In parallel, data‐driven modeling and machine learning approaches offer powerful tools to manage the nonlinear, history‐dependent behavior characteristics of volumetrically deforming soft materials, particularly in large‐area or high‐density arrays.

### 8.4 Morphological Programming and Scalable Fabrication

Geometry‐driven design strategies such as origami and kirigami offer a powerful means to encode function directly into morphology. Programmable anisotropy enables directed out‐of‐plane expansion, collapse, and friction modulation with minimal control overhead. Kirigami crawlers demonstrate anisotropic friction under inflation that supports directional motion through narrow conduits or across debris fields [287], while vacuum‐actuated foldable structures combine compact stowage with robust deployment for exploration tasks [233]. Origami metamaterial robots further illustrate adaptability to variable‐diameter pipes through expansion‐assisted anchoring and cleaning [288].

One of the persistent challenges in soft actuator research is that theoretical and computational models often fail to capture the full complexity of environmental interactions, including contact dynamics, frictional effects, material nonlinearities, hysteresis, and geometric imperfections. Some works have developed pressure feedback or parameter feedback models for position regulation of soft pneumatic actuators to improve control in 3D deformation. These are valuable but still limited when out‐of‐plane expansion involves contacting walls, deformable obstacles, or environmental uncertainty. In this context, computationally designed sensing architectures such as micro‐crumple or programmed‐crack structures offer promising routes toward scalable, deformation‐robust sensing [289, 290]. On the control side, comparative studies of hysteresis models provide guidance for compensation and trajectory tracking in soft pneumatic systems, yet real time performance in cluttered, contact‐rich environments remains limited [291].

At smaller scales, soft lithography and additive manufacturing enable dense arrays of out‐of‐plane actuators, yet packaging fluid routing, electrical isolation, sealing, and integration with electronics often becomes the bottleneck. Hybrid approaches that combine rigid islands for electronics with soft, stretchable interconnects and modular origami‐inspired units are gaining traction as a scalable compromise between manufacturability and actuation performance [292].

### 8.5 Biocompatibility and Safety for In‐Body and Human‐Centered Applications

For minimally invasive procedures, rehabilitation, and wearable systems, actuators must meet stringent requirements for biocompatibility, sterilizability, and safety. Multi‐material stacks common in soft actuators complicate standard sterilization processes, and uncontrolled expansion poses risks to surrounding tissue. Reviews of healthcare‐oriented soft devices emphasize the need for autoclavable elastomers, compatibility with ethylene oxide or plasma sterilization, and closed‐loop force limiting during expansion [293].

Future systems will benefit from in situ compliance and force sensing that enable real time modulation of expansion profiles to ensure tissue‐safe interaction. In such contexts, autonomy and safety are inseparable, making tight integration of actuation, sensing, and computation a critical design requirement rather than an optional enhancement.

### 8.6 Scalability, Manufacturing, and Deployment Readiness

Although thickness morphing has been demonstrated across length scales from millimeter‐scale haptic pixels to meter‐scale deployable structures scaling these systems while maintaining performance uniformity and reliability remains challenging. Fabrication complexity increases rapidly with actuator density, multilayer integration, and routing of electrical or fluidic networks. Moreover, variations introduced during manufacturing can lead to nonuniform deformation, drift, or failure under repeated use. Thickness‐morphing soft skins inherently operate across multiple length scales, and their performance is strongly influenced by scale‐dependent mechanisms. At the microscale, actuation strategies such as electroactive pixel arrays or localized hydrogel swelling enable precise control of surface topography and fine contact mechanics. At the milliscale, modular elements, including HASEL units and kirigami/origami‐inspired structures, translate localized deformation into coordinated motion and functional shape change. At larger scales, meter‐scale deployable skins rely on hierarchical assembly and structural integration to achieve macroscopic morphing and spatial coverage. Across these scales, key performance metrics, including actuation speed, force output, energy efficiency, mechanical robustness, and so forth, vary significantly, reflecting differences in underlying physical constraints and dominant deformation modes. As a result, effective design of thickness‐morphing systems requires explicit consideration of these scale‐dependent trade‐offs. Incorporating a multiscale perspective not only enables more systematic comparison of actuation strategies but also provides a framework for designing integrated soft skins that bridge microscale functionality with macroscale deployment.

Textile materials are also emerging as a versatile platform for soft morphing systems in human–machine interaction. Their inherent flexibility, conformability, and programmable architecture enable out‐of‐plane deformation while maintaining comfort and wearability. Recent studies have explored textile‐based metastructures for morphing robotics and bionic camouflage, demonstrating how fiber‐level design can achieve controlled local protrusions and surface reshaping [294, 295]. Incorporating textile‐structured materials offers a promising avenue for developing scalable, lightweight, and wearable thickness‐morphing skins, particularly for haptic interfaces, adaptive garments, and bioinspired robotics.

Further, lack of standardized metrics and testing protocols are lacking. Reported quantities such as “shrink ratio,” “stroke,” or “blocked force” are often measured under disparate boundary conditions, geometries, and environments, hindering meaningful comparison across studies. Many demonstrations emphasize peak performance under idealized laboratory conditions, while long‐term cycling, contamination exposure, and constrained operation are rarely explored. Establishing shared benchmarks covering expansion/shrinkage ratio, force output, response time, energy efficiency, durability cycles, and performance under confinement would significantly accelerate translation. Equally important is systematic reporting of failure modes and negative results, which remain underrepresented despite their critical relevance to real‐world deployment.

Progress toward deployment will require manufacturing‐aware design, favoring architectures compatible with scalable processes such as roll‐to‐roll fabrication, printing, lamination, and modular tiling. Morphological programming where geometry and material layout encode function offers a particularly powerful approach to reduce control complexity and tolerance sensitivity. Standardized testing protocols for thickness‐morphing performance, including metrics for out‐of‐plane stroke, force, bandwidth, efficiency, and lifetime, will also be essential for comparing technologies and accelerating translation beyond proof‐of‐concept demonstrations.

### 8.7 Toward Embodied Intelligence in Soft Skins

Looking forward, the most transformative impact of reversible thickness morphing may lie not in isolated actuation performance, but in enabling embodied intelligence at the material level. By dynamically redistributing volume, stiffness, and surface geometry, thickness‐morphing soft skins can physically mediate perception, interaction, and adaptation, reducing the burden on centralized sensing and computation. In this view, thickness change becomes a mechanism for encoding information, shaping interaction, and regulating behavior through morphology itself.

Realizing this vision will require convergent advances across materials science, soft mechanics, electronics, and control. Soft skins that autonomously sense contact, adapt shape, modulate impedance, and recover from damage would represent a qualitative shift from passive coverings or discrete actuators to active, perceptive material systems. Such systems could enable safer human–machine interfaces, adaptive prosthetics and wearables, resilient robots for unstructured environments, and responsive surfaces that dynamically negotiate their surroundings.

### 8.8 Outlook

Reversible thickness morphing emerges as more than a collection of actuation techniques it defines a materials‐centered design axis for adaptive interfaces and soft robotic systems. By consolidating diverse approaches under a unified framework grounded in surface‐normal deformation, this review delineates the current state of the art, clarifies fundamental trade‐offs, and identifies the materials and architectural strategies required to advance the field. Continued progress along these directions will be critical to transforming thickness‐morphing soft skins from compelling laboratory prototypes into autonomous, perceptive, and deployable technologies capable of operating reliably in real‐world environments.

The ability of skin to reversibly modulate thickness, curvature, and volume rather than merely bending or elongating opens new pathways for interaction, navigation, and adaptation in spaces where rigid mechanisms fail. For instance, social robots equipped with soft, muscle‐like facial skins can reproduce micro‐expressions such as wrinkles or smiles, improving emotional communication and human acceptance [296, 297]. Similarly, surface‐integrated e‐skins can provide visual cues, such as color change to reflect temperature or emotional states, or tactile feedback to indicate proximity or object interaction. The rapidly expanding fields such as internet of everything (IoE) [298], digital twins [299], and so forth, have further elevated the role of e‐skin as a multimodal communication medium between humans, machines, and environments.

Looking forward, the most transformative opportunity lies in self‐powered, computationally intelligent soft skins that tightly couple sensing, actuation, and morphology. As multifunctional materials, architected geometries, and hybrid machine‐learning‐physics approaches converge, thickness‐morphing soft actuators are poised not merely to emulate rigid systems, but to define a new class of adaptive, embodied machines whose capabilities emerge from the co‐design of softness, geometry, and intelligence.

## Conflicts of Interest

The authors declare no conflicts of interest.

## Data Availability

Data sharing is not applicable to this article as no new data were created or analyzed in this study.
